# Artificial-intelligence based prediction of flexible pavement condition deterioration using multi-temporal terrestrial laser scanning observations

**DOI:** 10.1038/s41598-026-68410-z

**Published:** 2026-09-11

**Authors:** Abdalziz Alruwaili, Ashraf A. A. Beshr, Hatem M. Elboraei, Sara Sameh

**Affiliations:** 1 Head of Civil Engineering Department, Department of Civil Engineering, College of Engineering, Jouf University, Sakaka, 72388 Kingdom of Saudi Arabia; 2 Head of Public Works Department, Faculty of Engineering, Mansoura University, Mansoura, 35516 Egypt; 3 General Director PES Company, Cairo, Egypt; 4 Faculty of Engineering and Technology, Badr University in Cairo (BUC), Cairo, Egypt

**Keywords:** Terrestrial laser scanning, PCI, Flexible pavement distress, Machine learning, Predictive maintenance, Engineering, Environmental sciences

## Abstract

In recent years, Egypt has witnessed a remarkable development in infrastructure projects as part of its Egypt Vision 2030, which aims to achieve sustainable development. Among the most prominent of these projects is the construction and development of efficient highway networks, which are a cornerstone for supporting transportation and connecting different governorates and cities. Given the vital role these roads play in economic and social development, monitoring their condition assessment and evaluating their efficiency regularly is crucial to improve traffic flow, enhancing road safety and reducing operating and maintenance costs. This paper investigates using Terrestrial Laser Scanning (TLS) as a high resolution, repeatable alternative for managing flexible pavement surface defects and develops a data-driven framework to predict pavement deterioration depending on several observations epochs. The objective is to quantify pavement surface distresses, track their temporal evolution, identify the distress characteristics most strongly associated with pavement condition loss, and forecast future pavement performance to support proactive maintenance planning. A 20.1 km roadway of the Kafr El-Sheikh city—Tanta city direction in Egypt was scanned with TLS instrument at eight positions and surveyed over four observations epochs (November 2022, July 2023, April 2024 and March 2025). Point clouds were processed to extract several distress types and the Pavement Condition Index (PCI) was computed for every roadway section and for all observations epochs. Multiple Linear Regression, Decision Tree, Random Forest, and XGBoost models were used in conjunction with correlation and regression analysis to assess distress–PCI relationships and forecast pavement condition. Across the studied roadway, PCI declined in every section with losses ranging from 5.4 to 45% over 28 months; pothole area was the fastest growing distress (up to + 45% per year). Correlation and regression analysis identified rutting, lane-to-shoulder drop-off and linear cracking as the distresses most strongly associated with reduced serviceability and a Random Forest model ranked raveling, linear cracking and rutting as the dominant PCI predictors. Per-section deterioration trend models (mean R^2^ = 0.91) forecast that sections 2 and 5 will reach structural failure (PCI < 25) before 2030. The paper's novelty is that it integrates repeated multi-temporal TLS observations, objective three-dimensional distress quantification, PCI-based assessment, statistical analysis, explainable machine-learning prediction, and long-term deterioration forecasting within a unified pavement-management framework. The results show that combining multi-temporal TLS observations with advanced statistical and machine-learning techniques can significantly improve pavement asset management by allowing for accurate distress quantification, reliable deterioration forecasting and proactive maintenance planning, ultimately improving roadway performance, safety and lifecycle sustainability.

## Introduction

Recently, Egypt has witnessed significant development in its roads and transportation sector, recognizing the importance of road networks in supporting sustainable development, raising living standards, and boosting economic growth. Highways and vital roads connecting different cities and governorates are among the most important infrastructure elements, facilitating the movement of people and goods and achieving developmental integration between regions. To ensure the continued efficiency of these roads and maintain their service level, it has become essential to monitor their structural condition and evaluate their performance periodically by identifying and addressing defects and deterioration that may appear over time^1,2^. Technological advancements have led to the emergence of numerous modern devices and technologies that offer more accurate and efficient alternatives to traditional visual inspection methods^3–5^. These technologies enable the early detection of defects and a comprehensive and objective assessment of pavement condition^3,6^. Relying on these modern technologies contributes to supporting road management and maintenance operations by prioritizing maintenance and selecting the most appropriate intervention methods at the right time. This helps extend the lifespan of roads, reduce future maintenance costs, and prevent the worsening of defects, which positively impacts traffic safety and the efficiency of the transportation network.

Road networks are among the most valuable public assets, and once surface distresses are allowed to spread into the structural layers, pavement maintenance becomes extremely expensive. Therefore, early detection and quantification of problems, monitoring their evolution, and predicting when a section will drop below an acceptable level of service are essential for effective pavement management^7–10^. Conventional management is based on routine visual inspection, whereby skilled surveyors drive or walk a route and use standardized methods to grade distresses by eye^11,12^. Although this method is cheap, it is subjective, exposes employees to real-time traffic, only records a portion of the pavement surface, and, as this study's field data demonstrates, frequently records significant distresses as “cannot be measured.”

Terrestrial Laser Scanning (TLS) provides an objective, contactless, and highly repeatable alternative^1,2^. A TLS instrument records millions of three-dimensional points each scan, resulting in a dense surface model from which cracking length, raveling and patching regions, rutting depth, and pothole shape may be measured directly and later premeasured with sub-centimeter precision^1,4^. Comparative studies have demonstrated that laser-based approaches can accurately detect medium- and high-severity distresses, as well as quantify crack area and volume, as compared to manual surveys^1,3^. Because each survey is geo-referenced, repeated scans may be stacked to measure how each distress evolves over time, which is critical for degradation models but nearly hard to get by sight^6^. Laser scanning has been shown to be a reliable source of pavement-condition data by a large body of research. Rut depth has been measured from cross-sectional profiles using mobile and terrestrial laser scanning^13,14^, surface distress has been assessed from dense point clouds^15^, and cracks have been directly detected and clustered in three-dimensional data using region-growing, graph-convolutional, and time-grid algorithms^5,16,17^. Point-cloud metrology can measure fracture breadth, length and continuity, pothole depth and volume, and rut profiles and map these characteristics onto agency frameworks like the PCI, according to reviews of three-dimensional imaging^18^.

Simultaneously, data-driven deterioration modeling has advanced quickly: deep-learning segmentation now automatically classifies distresses from three-dimensional surface images^19,20^, decision-tree and tree-ensemble methods have been used to predict PCI from historical distress data^21–23^, back-propagation and deep neural networks have been used to estimate PCI and related indices^24,25^. While stressing that their transferability is limited by small or spatially clustered samples, these studies consistently find that machine-learning models can match or surpass conventional regression when trained on large datasets^26,27^.

Although recent studies have demonstrated the potential of using terrestrial laser scanning for pavement distress measurement and machine-learning algorithms for pavement condition prediction, most investigations rely on single-epoch surveys or historical distress databases. Consequently, they cannot explicitly capture the temporal evolution of pavement deterioration under real operating conditions. Furthermore, only limited research has integrated repeated TLS observations with explainable statistical analysis and machine-learning models within a unified pavement management framework capable of both distress quantification and long-term condition forecasting.

Despite this advancement, few studies have combined repeated, multi-temporal terrestrial laser scanning of the same corridor with a transparent statistical and machine-learning technique that quantifies the PCI-distress relationship while also forecasting future conditions. This paper fills that gap. It combines four epochs of TLS observations with statistical and machine-learning analysis to provide a comprehensive management workflow, including distress quantification, deterioration rate estimation, identification of the distresses that most strongly drive condition loss, prediction of the PCI to 2030, and a risk-based maintenance strategy. The novelty of this study lies in integrating four multi-temporal TLS surveys, objective three-dimensional distress quantification, PCI computation, statistical deterioration analysis, explainable machine-learning models, and long-term pavement deterioration forecasting into a unified decision-support framework. Unlike previous studies that primarily focus on either pavement distress detection or condition prediction, the proposed framework simultaneously quantifies pavement deterioration, identifies the dominant distress mechanisms governing PCI degradation, forecasts future pavement conditions, and supports proactive maintenance prioritization. Additionally, it illustrates the usefulness of TLS in identifying and precisely quantifying pavement distresses that are not consistently measured by traditional visual inspection, allowing for data-driven and proactive repair decision-making. Therefore, the objectives of this research can be summarized as following:

1. Develop a framework to automatically extract and quantify flexible pavement surface distresses from TLS point clouds and compare it with conventional visual inspection.
2. Monitor the evolution of each distress and the PCI as a whole throughout the course of four observations epochs (2022–2025).
3. Quantify each road section's pavement deterioration rate.
4. Use regression, correlation and machine-learning importance analysis to determine which types of distress have the most impact on PCI.
5. To assist with proactive maintenance planning, forecast future pavement conditions at 2030 and categorize the road into risk groups.

By integrating high resolution multi-temporal TLS observations with statistical analysis and explainable machine learning models, the proposed framework provides a practical and data-driven approach for continuous pavement condition monitoring, deterioration assessment and long-term maintenance planning. The developed methodology is designed to help transportation authorities improve maintenance prioritization, resource allocation, and road infrastructure sustainability and serviceability.

## Study area, instruments and data acquisition

### Study roadway

**Fig. 1:**
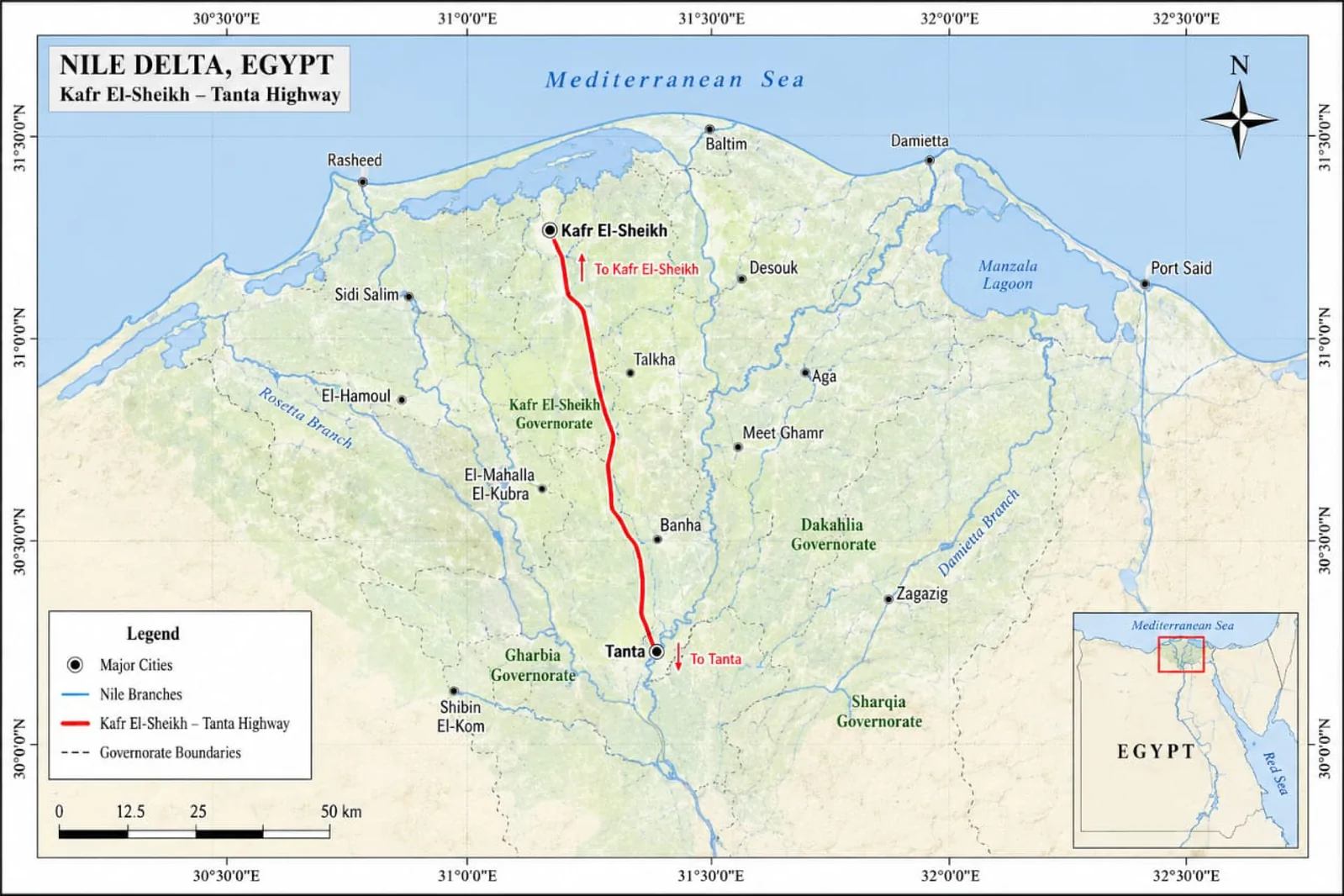
Location of the Kafr El-Sheikh–Tanta roadway in Nile Delta, Egypt (The map was prepared by the authors using ArcGIS Desktop 10.8 (Esri, Redlands, CA, USA). https://www.esri.com/en-us/arcgis/products/arcgis-desktop/overview)

The study area is located in the central Nile Delta of Egypt along the regional roadway connecting Kafr El-Sheikh and Tanta, two major cities that play important roles in the agricultural, commercial, industrial, and transportation sectors of the Delta region. Kafr El-Sheikh serves as the administrative capital of Kafr El-Sheikh Governorate and is well known for its agricultural production and fish farming activities, whereas Tanta, the capital of Gharbia Governorate, is a major commercial and industrial center that connects several neighboring governorates. Consequently, the roadway linking these two cities represents one of the principal regional transportation corridors in the Nile Delta, supporting the daily movement of passengers, agricultural products, and commercial goods (Fig. 1). The investigated roadway segment is approximately 20.1 km long and passes through the flat alluvial terrain that characterizes the Nile Delta. Owing to its strategic importance, the roadway experiences continuous traffic from heavy trucks, buses, private vehicles, and seasonal agricultural machinery. The combination of frequent heavy axle loads, intensive traffic operations, and environmental conditions such as temperature fluctuations and groundwater effects has contributed to the development of a wide range of pavement distresses, including longitudinal and transverse cracking, rutting, potholes, raveling, and localized surface deformation. These characteristics make the study corridor particularly suitable for evaluating pavement deterioration under actual operating conditions. The coexistence of different distress types and severity levels provides an opportunity to assess the capability of terrestrial laser scanning (TLS) to detect, quantify, and monitor pavement deterioration with high spatial resolution. Furthermore, repeated surveys conducted over multiple observation epochs enable the temporal evolution of pavement condition to be investigated, providing a reliable basis for deterioration analysis and predictive modelling. The selected roadway therefore provides a representative environment for integrating high-resolution TLS observations with statistical analysis and machine-learning techniques to evaluate pavement condition, identify the dominant deterioration mechanisms, and support long-term maintenance planning. The diversity of traffic conditions, pavement distress mechanisms, and environmental influences within a single transportation corridor enhances the applicability of the proposed methodology to similar regional road networks.

**Fig. 2:**
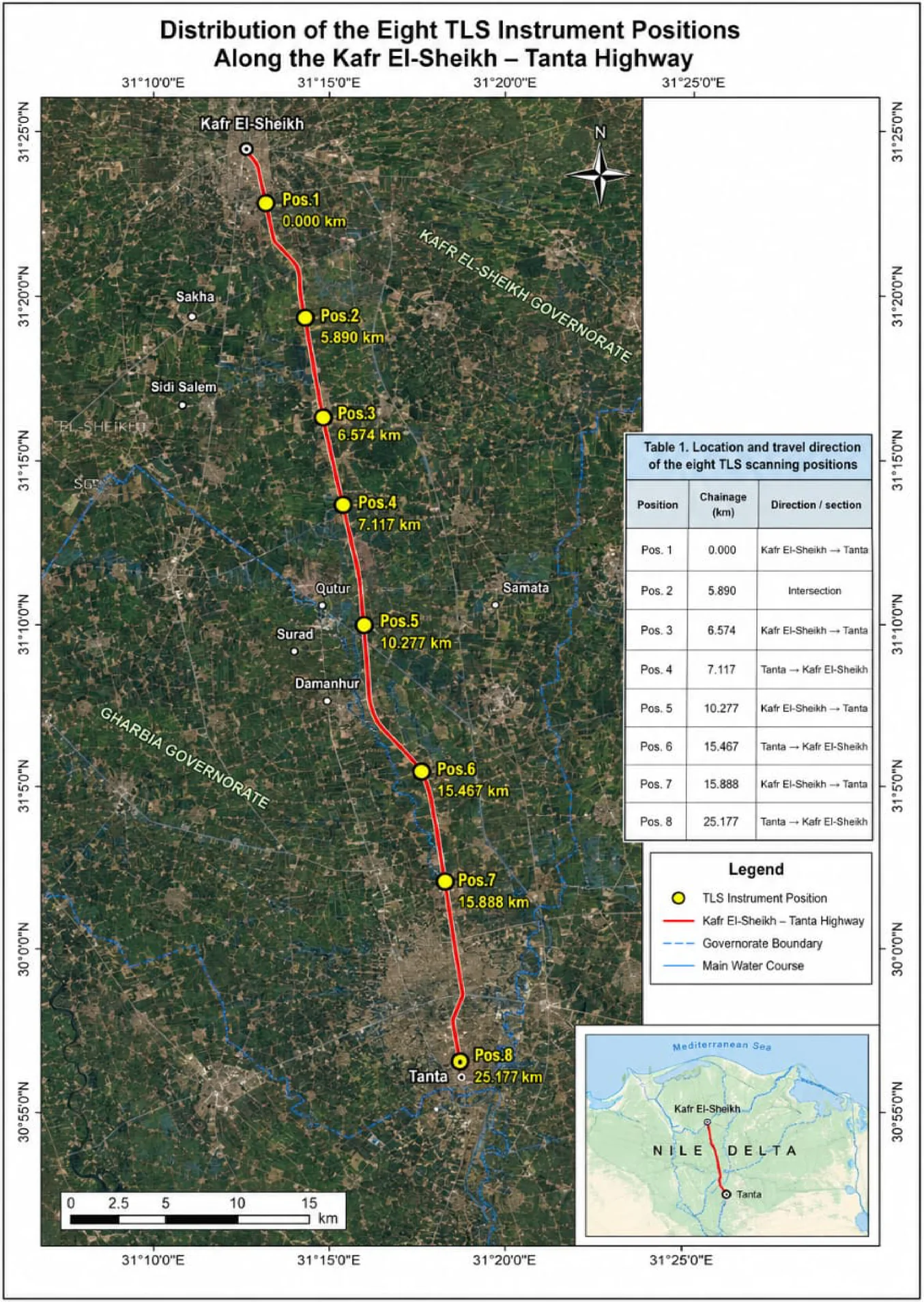
Distribution of the eight TLS instrument positions along the studied roadway (Satellite imagery was obtained from Copernicus Sentinel-2 data, and the map layout and thematic information were prepared by the authors using ArcGIS Desktop 10.8 (Esri, Redlands, CA, USA)

Eight scanning positions were distributed along the studied roadway to capture representative sections in both directions. Figure 2 shows the eight instrument positions, and Table 1 lists their chainage and travel direction. The stations occupied by TLS for road surface data collection were clearly identified and established at the first time observations (first epoch—November 2022) were taken. These stations were identified and labeled for easy access each subsequent monitoring epoch.

**Table 1:** Location and travel direction of the eight TLS instrument positions for all observations epochs.

Position	Roadway chainage (km)	Direction/section
Pos. 1	0.000	Kafr El-Sheikh → Tanta
Pos. 2	5.890	Intersection
Pos. 3	6.574	Kafr El-Sheikh → Tanta
Pos. 4	7.117	Tanta → Kafr El-Sheikh
Pos. 5	10.277	Kafr El-Sheikh → Tanta
Pos. 6	15.467	Tanta → Kafr El-Sheikh
Pos. 7	15.888	Kafr El-Sheikh → Tanta
Pos. 8	20.125	Tanta → Kafr El-Sheikh

### TLS Instrument and scan configuration

**Fig. 3:**
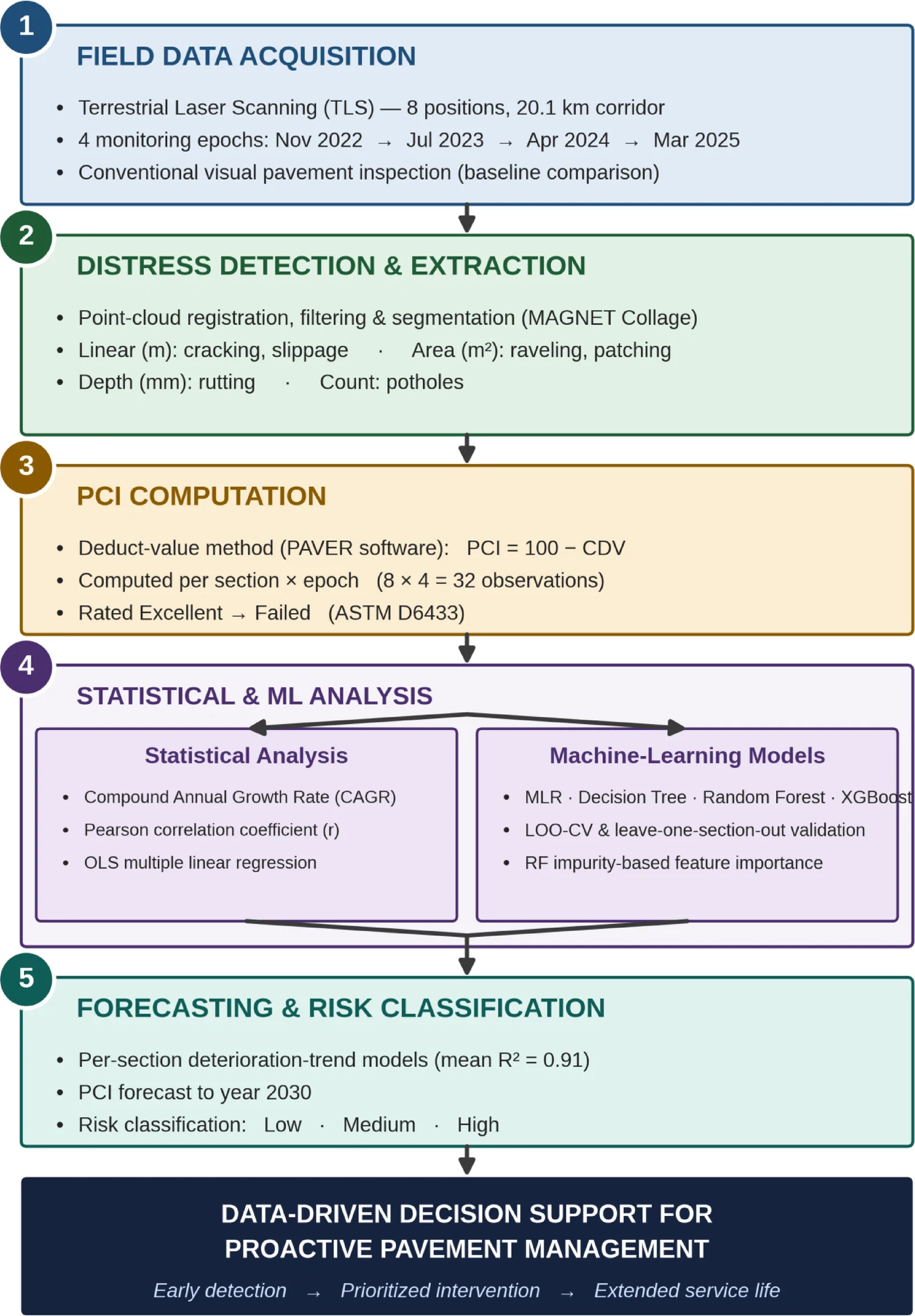
Flowchart for research plan and methodology

A Topcon GLS-2000 terrestrial laser scanner (TLS), which produces high resolution 3D point cloud data appropriate for pavement distress evaluation, was used to evaluate the pavement condition assessment. The scanning distance of the used laser scanner can be up to 350 m from the target. The distance accuracy of TLS is 2 mm (for a distance of 150 m). It takes around 18 min to obtain more than 5 million scanned point clouds with a 6 s angular resolution. The compensation range is ± 6 min, with a target detection precision of 3 s at 50 m (Fig. 3).

### Multi-temporal survey design

Eight scanner positions were occupied along the study roadway, which cover the whole length of 20.1 km long between Kafr El-Sheikh city and Tanta city. In order to guarantee sufficient and complete coverage of the pavement surface and capture various kinds of surface defects, ten scans were carried out at the specified positions. The distance between the scanner and the road surface was taken into consideration when choosing the scan resolution. While lower resolutions were adequate for overall route coverage, higher resolutions were utilized in regions that required precise assessments of pavement distresses. All the same field procedures were done at each observations epoch (November 2022, July 2023, April 2024 and March 2025). To ensure the consistency of the temporal analysis, identical scanning parameters, scanner locations, acquisition geometry and field procedures were maintained throughout all observation epochs. This minimized systematic measurement variations between surveys and ensured that the detected changes represented actual pavement deterioration rather than differences in data acquisition conditions. Such consistency is essential for reliable long-term monitoring using terrestrial laser scanning^28,29^.

The scan resolutions varied between 1.2 mm at 10 m and 3.1 mm at 40 m. Higher resolution scans necessitated longer acquisition time, as was to be expected. All 3D point clouds were entered into the MAGNET Collage program for processing and analysis all data collection. Sub-clouds representing distinct pavement portions were created by registering, filtering and dividing the point clouds. Pavement surface distresses were then measured and identified using these datasets and their evaluation over the various observation epochs was assessed. Table 2 displays the attributes of the ten scans such as scan mode, resolution, duration and chainage TLS position.

**Table 2:** Properties of the ten TLS scans at eight positions fixed for all observations epochs.

Scan number	Mode of scan	Resolution/distance	Scan time (min)	Chainage (km)
First	Road	3.1 mm/40 m	1.53	0.000 (Kafr El-Sheikh)
Second (1)	–	1.6 mm/25 m	3.43	5.890
Second (2)	Standard	1.2 mm/10 m	3.24	5.890
Second (3)	Standard	–	9.50	5.890
Third	Road	1.6 mm/25 m	6.34	6.574
Fourth	–	3.1 mm/40 m	2.50	7.117
Fifth	–	–	7.00	10.277
Sixth	–	1.6 mm/25 m	5.59	15.467
Seventh	–	–	6.46	15.888
Eighth	–	–	6.45	20.125

The selected scan resolutions provided an appropriate balance between geometric accuracy, acquisition time, and computational efficiency. High-resolution scans were adopted for detailed pavement distress quantification, whereas lower resolutions were sufficient for corridor-scale coverage. This scanning strategy enabled the generation of dense and accurate point clouds while maintaining practical field survey duration for repeated observations.

The multi-temporal TLS datasets generated during the four monitoring epochs formed the primary database for all subsequent pavement distress quantification, statistical analysis, machine-learning modeling and deterioration forecasting presented in this study. The proposed methodological framework follows established principles of pavement condition assessment using terrestrial laser scanning while extending previous studies through the integration of repeated TLS observations and predictive machine learning analysis for long-term deterioration modeling^29–31^.

### Conventional visual inspection

**Fig. 4:**
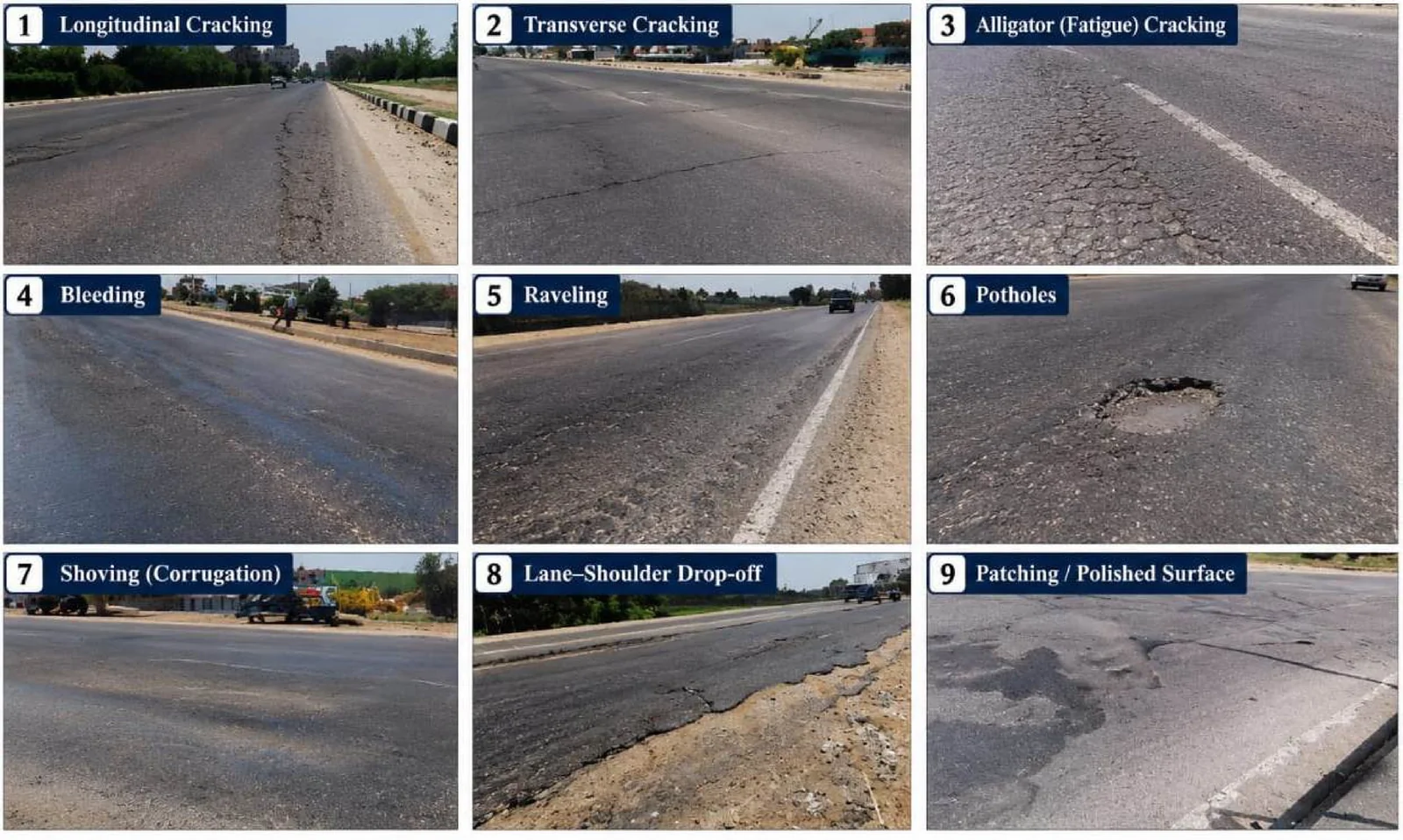
Representative pavement surface condition and major distress types identified during the field inspection campaign (July 2023)

**Fig. 5:**
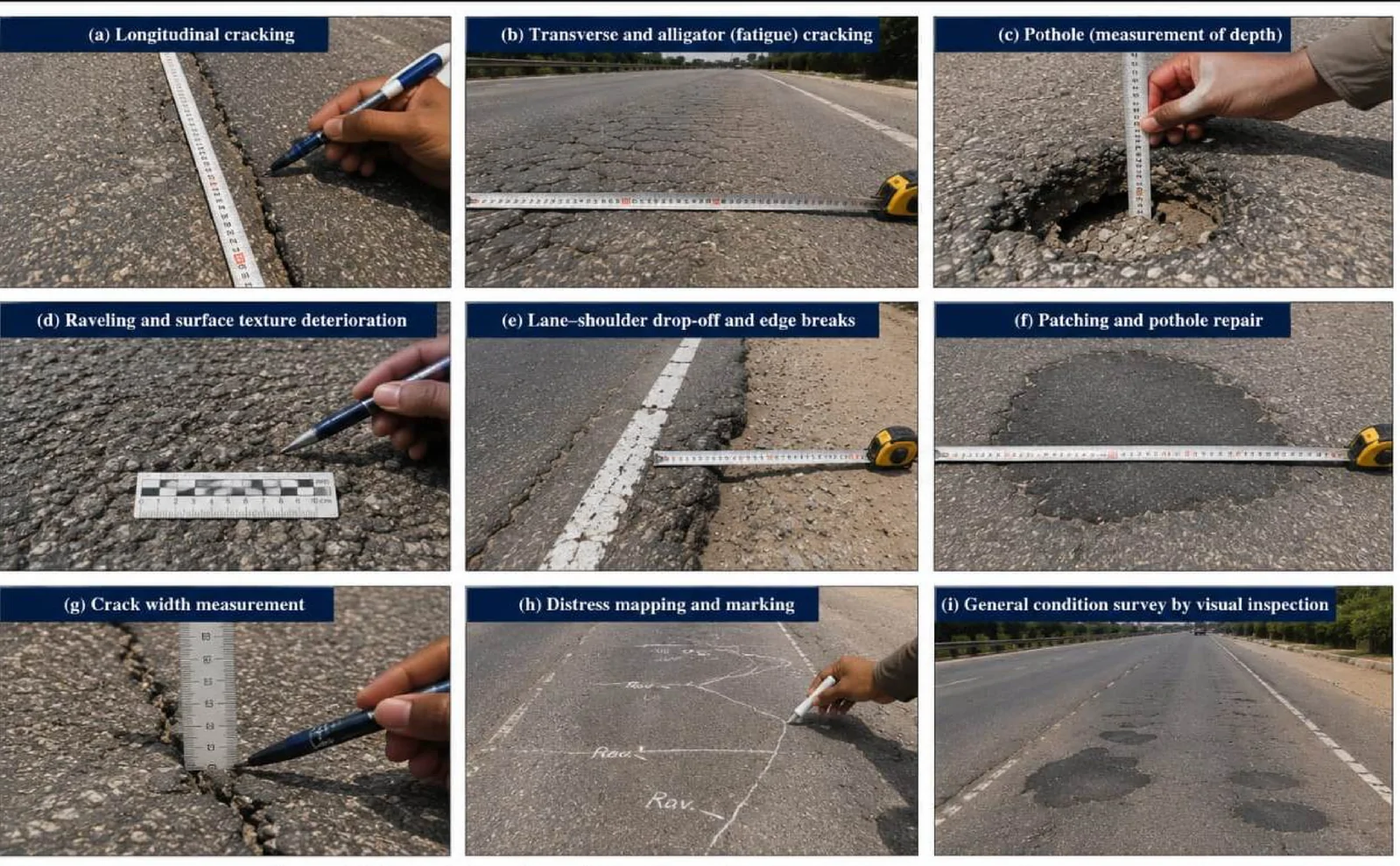
Conventional visual inspection and manual measurement of representative pavement distresses used for validation of TLS-derived measurements

A conventional visual inspection survey was initially conducted to establish a reference condition and provide a basis for comparison with the TLS-derived pavement distress measurements. Figure 4 presents the general condition of the investigated roadway, while Fig. 5 illustrates representative pavement distresses identified during the field survey conducted in July 2023, including fatigue (alligator) cracking, block cracking, potholes, edge break-up, and slippage cracking. Where the distresses were safely accessible, manual measurements were collected using a tape measure and measuring rule to provide reference values for comparison with the TLS-derived measurements (Fig. 5). However, several pavement distresses could not be safely accessed or accurately measured manually due to their location, extent, or severity. These limitations highlight the advantage of TLS technology, which enables contactless and comprehensive measurement of pavement surface characteristics while minimizing field safety risks.

The combination of visual inspection and TLS measurements provided complementary information throughout the study. While visual inspection remains effective for rapid field identification of visible pavement defects, TLS offers objective geometric measurements with millimeter level accuracy and enables comprehensive documentation of the pavement surface. Moreover, the resulted digital 3D point cloud database produced by TLS can be reanalyzed in the future without requiring additional field visits, providing a significant advantage for long-term pavement monitoring and infrastructure asset management^29,32^.

Although terrestrial laser scanning provides highly accurate three-dimensional measurements, its effectiveness depends on appropriate field conditions, including adequate scanner positioning, unobstructed visibility, and proper registration of successive scans. These considerations were carefully addressed during all field campaigns to ensure consistent data quality across the four observation epochs.

## Research methodology and plan

The main procedure and plan used in this study to evaluate and assessment pavement conditions, analyze deterioration and forecast the highway's future performance is shown in Fig. 3. There are five primary stages to the research methodology. First, several epochs of terrestrial laser scanning (TLS) observations and traditional visual inspection were carried out to collect field data throughout four monitoring epochs from 2022 to 2025. Second, 3D point clouds obtained from the TLS survey were used to identify and quantify several forms of pavement surface defects and distresses, such as erosion, potholes, cracks and craters. Third, the PAVER system was utilized to compute the PCI for every road section and for every observations epoch using the collected defect data. Fourth, in order to determine the most significant defect indicators and evaluate their influence on the PCI, statistical studies and machine learning techniques were used to examine the relationship between pavement flaws and their deterioration. Lastly, a decision-support framework for proactive and economical pavement maintenance management was created by modeling deterioration trends to forecast future pavement conditions at year 2030 and categorize road segments based on their degree of risk. The flowchart describing the exact procedure for research plan of the study can be found in Fig. 3.

## Integrated data processing and analysis

This section describes the procedures used to analyze the multi-temporal TLS observations. After processing the point clouds, pavement surface distresses were identified and measured quantitatively for each observation epoch. These measurements were used to calculate the PCI for every roadway section. The resulting database was then analyzed to investigate deterioration trends, examine the relationship between individual distress types and PCI, identify the most influential distress variables and predict future pavement condition.

### Detecting roadway defects from TLS observations (3D point clouds)

Point clouds collected during each observation epoch were imported into MAGNET Collage software for registration, geo-referencing, and pavement surface analysis. Dense three-dimensional point clouds that depict the pavement surface and its surrounding characteristics are obtained using the terrestrial laser scanner (TLS). The scanner was calibrated prior to field data collection in order to confirm its operating precision and guarantee the calibre and dependability of the readings obtained. MAGNET Collage, which offers a three-dimensional depiction of the surveyed pavement surface and makes it easier to see and quantify pavement surface flaws, was used to process the gathered point-cloud data. The geometric features and spatial extent of pavement distresses can be immediately identified and measured from the three-dimensional data thanks to the dense point-cloud representation. The observed pavement distresses could be quantitatively compared over time thanks to the consistent application of this approach to the TLS data from the four monitoring epochs. After registration, the pavement surface was segmented and individual distress types were identified and measured directly from the three-dimensional point clouds. The same processing procedure was applied to all observation epochs to ensure that distress measurements were directly comparable throughout the monitoring period. Total length (m) was used to quantify linear distresses, including longitudinal, transverse, and slipping cracking. Fatigue cracking, block cracking, raveling, patching, and bleeding were among the area-related distresses that were identified and measured in square meters (m2). Elevation profiles taken from the point clouds were used to assess surface deformation distresses, and their maximum depths were expressed in millimeters. Potholes were located and measured based on their quantity and impacted area. All TLS datasets were referenced to the same coordinate system, allowing the same pavement sections to be evaluated during each survey. This made it possible to monitor the progression of individual distress types over time and to calculate deterioration rates for each roadway section. The resulting measurements formed the basis for PCI computation, statistical analysis, and deterioration prediction presented in the following sections.

### PCI computation and distress condition relationship

The PCI was used as the primary indicator of pavement condition and serviceability. For every section and epoch the extracted distress quantities, types and severities were entered into the PAVER pavement-management system^33^ to compute the PCI.

The PCI is a numerical index ranging from 0 to 100, where higher values indicate better pavement condition and lower maintenance requirements defined in ASTM D6433^12,34^. PCI bands were interpreted as Excellent (> 85), Good (70–85), Fair (55–70), Poor (40–55), Very Poor (25–40) and Failed (< 25). This classification provides a practical basis for evaluating pavement performance and prioritizing maintenance interventions.

The PCI is calculated from the type, severity, and extent of the observed pavement distresses using the deduct value procedure defined in ASTM D6433. Each distress contributes a deduct value according to its severity and density. The individual deduct values are combined to obtain the corrected deduct value (CDV), and the PCI is calculated as:

1 $$$$$$ {\mathrm{PCI}} = {1}00 - {\mathrm{CDV}} $$$$$$

where CDV = f (DV₁, DV₂, …, DV_n_), in which CDV is represents the corrected cumulative impact of all identified distresses within the pavement section and DV_i_ is the deduct of the i-th distress.

Because every measured distress; cracking, raveling, rutting, potholes, bleeding and so on adds a deduct, increasing distress quantity or severity necessarily lowers PCI. This is precisely why the dense, repeatable distress quantities produced by TLS are valuable: they feed the deduct-value engine with objective inputs, and they make it possible to study, statistically, which distresses contribute most to the loss of PCI. The correlation, regression and machine-learning analyses that follow quantify this PCI distress relationship empirically from the field data.

## Predictive modeling framework

### Statistical analysis and the regression principle

The statistical analyses were performed using the TLS-derived distress measurements collected from eight roadway sections over four observation epochs, resulting in a dataset of 32 section–epoch observations. Three complementary statistical methods were employed to quantify pavement deterioration, investigate the relationship between pavement distresses and PCI, and identify the distress variables that most strongly influence pavement condition as in Table 3. These analyses also provide the statistical basis for the machine-learning models presented in the following section.

**Table 3:** Summary of statistical analyses.

Analysis	Purpose	Output
Compound Annual Growth Rate (CAGR)	Quantify annual deterioration of each distress	Annual growth rate (%)
Pearson correlation	Measure association between distress variables and PCI	Correlation coefficient (r)
Multiple Linear Regression (OLS)	Quantify the contribution of each distress to PCI	Regression coefficients, R^2^, RMSE

Each statistical method provides complementary information. CAGR describes deterioration over time, Pearson correlation evaluates the strength of the relationship between individual distress types and PCI, and multiple regression estimates the combined influence of all distress variables on pavement condition.

#### Compound annual growth rate (CAGR)

The Compound Annual Growth Rate (CAGR) was used to estimate the average annual rate of deterioration for each pavement distress over the monitoring period. Unlike absolute changes, CAGR expresses deterioration as an equivalent annual rate, allowing different distress types to be compared on a common basis. This approach is particularly useful when observations span multiple years because it accounts for differences in the duration of the monitoring period^35^. The CAGR was calculated as in Eq. (2):

2 $$$$$$ {\text{CAGR = }}\left( {\frac{{V_{f} }}{{V_{i} }}} \right)^{\frac{1}{t}} - 1 $$$$$$

where $$$$$${V}_{i}$$$$$$ is the measured quantity of a pavement distress during the initial observation epoch; $$$$$${V}_{f}$$$$$$ is the measured quantity of the same distress during the final observation epoch; and *t* is the monitoring period expressed in years.

Positive CAGR values indicate progressive deterioration, whereas values close to zero indicate relatively stable conditions. Higher values correspond to faster deterioration rates and therefore identify pavement distresses that may require earlier maintenance. CAGR was selected because it provides a normalized measure of deterioration that facilitates comparison among different pavement distress types despite differences in their initial quantities and units of measurement.

#### Pearson correlation analysis

Pearson's correlation analysis was performed to evaluate the linear relationship between each pavement distress variable and the PCI. This analysis provides an initial assessment of the strength and direction of the association between individual distress types and pavement condition. Identifying these relationships helps determine which distress variables are most strongly associated with PCI deterioration before developing multivariate regression and machine-learning models^36^. The Pearson correlation coefficient was computed using the classical formulation proposed by^37^. The Pearson correlation coefficient (r) was calculated as follows Eq. (3):

3 $$$$$$ r = \frac{{\sum\nolimits_{i = 1}^{n} {\left( {x_{i} - \overline{x}} \right)\left( {y_{i} - \overline{y}} \right)} }}{{\sqrt {\sum\nolimits_{i = 1}^{n} {\left( {x_{i} - \overline{x}} \right)^{2} \sqrt {\sum\nolimits_{i = 1}^{n} {\left( {y_{i} - \overline{y}} \right)^{2} } } } } }} $$$$$$

where $$$$$${x}_{i}$$$$$$ represents the observed value of a pavement distress variable; $$$$$${y}_{i}$$$$$$ represents the corresponding PCI; $$$$$$\overline{x}$$$$$$ and $$$$$$\overline{y}$$$$$$ are the sample means of the distress variable and PCI, respectively; *n* is the total number of observations.

The correlation coefficient ranges from − 1 to + 1. A value close to − 1 indicates a strong negative linear relationship, a value close to + 1 indicates a strong positive linear relationship, whereas values near 0 indicate little or no linear association. The statistical significance of each correlation was evaluated using the corresponding *p*-value, with *p* < 0.05 considered statistically significant. It should be noted that correlation analysis evaluates the relationship between two variables independently and does not account for the simultaneous influence of other distress variables. Therefore, Pearson's correlation analysis was complemented by multiple linear regression to assess the combined effects of the measured pavement distresses on PCI.

#### Ordinary least squares (OLS) regression

Ordinary Least Squares (OLS) multiple regression was used to quantify the relationship between the measured pavement distress variables and the Pavement Condition Index (PCI). Unlike Pearson's correlation analysis, which evaluates the relationship between two variables independently, OLS regression estimates the contribution of each distress variable while simultaneously accounting for the influence of the remaining predictors. This provides a comprehensive assessment of the factors controlling pavement deterioration and enables the identification of the pavement distress variables that most strongly influence PCI^38^. The multiple linear regression model is expressed as in Eq. (4):

4 $$$$$$\mathrm{y}=\mathrm{X}\upbeta +\upvarepsilon $$$$$$

where $$$$$$\mathbf{y}$$$$$$ is the vector of observed PCI values; $$$$$$\mathbf{X}$$$$$$ is the design matrix containing the measured pavement distress variables; $$$$$${\boldsymbol{\upbeta}}$$$$$$ is the vector of regression coefficients to be estimated; $$$$$${\boldsymbol{\upvarepsilon}}$$$$$$ is the vector of random errors.

The regression coefficients were estimated using the Ordinary Least Squares Estimator Eq. (5):

5 $$$$$$\widehat{\upbeta }={\left({\mathrm{X}}^{T}\mathrm{X}\right)}^{-1}{\mathrm{X}}^{T}\mathrm{y}$$$$$$

where $$$$$$\widehat{{\boldsymbol{\upbeta}}}$$$$$$ is the vector of estimated regression coefficients and $$$$$${\mathbf{X}}^{T}$$$$$$ denotes the transpose of the design matrix.

The estimator in Eq. (5) is obtained by minimizing the residual sum of squares between the observed and predicted PCI values. The least-squares objective function is defined as in Eq. (6)^39,40^

6 $$$$$$S\left(\upbeta \right)=\sum_{i=1}^{n}{\left({y}_{i}-{\widehat{y}}_{i}\right)}^{2}={\left(\mathrm{y}-\mathrm{X}\upbeta \right)}^{T}\left(\mathrm{y}-\mathrm{X}\upbeta \right)$$$$$$

where $$$$$$S\left({\boldsymbol{\upbeta}}\right)$$$$$$ is the residual sum of squares, $$$$$${y}_{i}$$$$$$ is the observed PCI, and $$$$$${\widehat{y}}_{i}$$$$$$ is the predicted PCI.

Minimizing Eq. (6) with respect to the regression coefficients produces the normal equations^40^.

7 $$$$$${\mathrm{X}}^{T}\mathrm{X}\widehat{\upbeta }={\mathrm{X}}^{T}\mathrm{y}$$$$$$

whose solution corresponds to the estimator presented in Eq. (5). The variance of the regression residuals was estimated as Eq. (8)

8 $$$$$${\widehat{\sigma }}^{2}=\frac{S\left(\widehat{\upbeta }\right)}{n-k-1}$$$$$$

where *n* is the total number of observations and *k* is the number of predictor variables. This quantity represents the variance of the unexplained component of the regression model^39^. The uncertainty associated with each estimated regression coefficient was evaluated using its standard error Eq. (9):

9 $$$$$$SE\left({\widehat{\beta }}_{j}\right)=\sqrt{{\widehat{\sigma }}^{2}{\left({\mathrm{X}}^{T}\mathrm{X}\right)}_{jj}^{-1}}$$$$$$

where $$$$$${\left({\mathbf{X}}^{T}\mathbf{X}\right)}_{jj}^{-1}$$$$$$ is the *j*-th diagonal element of the inverse information matrix^40^.

The statistical significance of each regression coefficient was assessed using Student’s *t*-statistic as in Eq. (10)

10 $$$$$${t}_{j}=\frac{{\widehat{\beta }}_{j}}{SE\left({\widehat{\beta }}_{j}\right)}$$$$$$

where larger absolute values of $$$$$${t}_{j}$$$$$$ indicate stronger statistical evidence that the corresponding predictor contributes significantly to the regression model. The overall explanatory power of the regression model was evaluated using the coefficient of determination (R^2^) as in Eq. (11)

11 $$$$$${R}^{2}=1-\frac{S{S}_{res}}{S{S}_{tot}}$$$$$$

where $$$$$$S{S}_{res}$$$$$$ is the residual sum of squares and $$$$$$S{S}_{tot}$$$$$$ is the total sum of squares.

Higher $$$$$${R}^{2}$$$$$$ values indicate that a greater proportion of the variability in PCI is explained by the regression model^39^. In addition to $$$$$${R}^{2}$$$$$$, model performance was evaluated using the adjusted coefficient of determination (Adjusted $$$$$${R}^{2}$$$$$$), Root Mean Square Error (RMSE), the F-test, and the *p*-values of the regression coefficients. These statistical measures collectively assess the explanatory power, prediction accuracy, and overall significance of the fitted model. Before interpreting the regression results, the assumptions of linearity, independence, homoscedasticity, and normality of residuals were verified using residual diagnostic analysis^39^. The statistical indicators used to evaluate the regression model are summarized in Table 4.

**Table 4:** Statistical indicators used to evaluate the regression model.

Indicator	Description	Interpretation
$$$$$${R}^{2}$$$$$$	Coefficient of determination	Proportion of variance in PCI explained by the regression model
Adjusted $$$$$${R}^{2}$$$$$$	Adjusted coefficient of determination	Corrects $$$$$${R}^{2}$$$$$$ for the number of predictor variables
RMSE	Root Mean Square Error	Average prediction error of the regression model
F-test	Overall model significance	Evaluates whether the regression model is statistically significant
*p*-value	Predictor significance	Indicates whether an individual regression coefficient differs significantly from zero

The statistical indicators presented in Table 4 were jointly used to evaluate model performance. While R^2^ and Adjusted R^2^ quantify the explanatory capability of the model, RMSE measures prediction accuracy, and the F-test together with the *p*-values evaluates the statistical significance of both the overall regression model and the individual predictor variables.

### Machine learning models

Machine-learning models were developed to predict the PCI using pavement distress variables extracted from the multi-temporal TLS observations. Unlike conventional statistical methods, which generally assume linear relationships between predictors and the response variable, machine-learning algorithms can capture complex interactions and nonlinear deterioration patterns. In this study, machine learning was employed to (i) compare the predictive performance of different regression algorithms and (ii) identify the pavement distress variables that contribute most significantly to pavement deterioration. The statistical analyses presented in Section “5.3” were used to support the interpretation of the machine-learning results.

#### Model development

The predictor variables consisted of the pavement distress measurements extracted from the TLS point clouds, while the PCI calculated using the PAVER pavement management system according to ASTM D6433, was used as the response variable. Each observation represented one roadway section at one monitoring epoch, resulting in a dataset of repeated observations collected throughout the study period. To ensure a fair comparison, the same predictor variables were used for all evaluated machine-learning models^41,42^

#### Model training and validation

Model performance was evaluated using the coefficient of determination R^2^ and the RMSE, which quantify the goodness-of-fit and prediction accuracy of each regression model, respectively. Because the available dataset consisted of repeated observations from eight roadway sections, Leave-One-Out Cross-Validation (LOO-CV) was adopted to maximize the use of the available data while reducing the effect of the relatively small sample size. In addition, Leave-One-Section-Out (LOSO) validation was performed to assess the ability of the developed models to predict pavement condition for roadway sections that were excluded during model training, thereby providing a more rigorous evaluation of model generalization^43,44^.

#### Evaluated regression algorithms

Four supervised regression algorithms were evaluated in this study: Multiple Linear Regression (MLR), Decision Tree (DT), Random Forest (RF), and Extreme Gradient Boosting (XGBoost). These algorithms were selected because they represent different levels of model complexity, ranging from conventional linear regression to ensemble learning methods capable of modelling nonlinear relationships between pavement distress characteristics and pavement condition^45,46^. Previous studies have shown that tree-based ensemble methods generally outperform conventional regression models when modelling complex pavement deterioration processes^47–49^. A summary of the evaluated algorithms is provided in Table 5.

**Table 5:** Machine learning algorithms evaluated in this study.

Model	Characteristics	Main advantage
Multiple Linear Regression (MLR)	Linear regression	Simple and interpretable baseline model
Decision Tree (DT)	Tree-based regression	Captures nonlinear relationships with straightforward interpretation
Random Forest (RF)	Ensemble learning	High prediction accuracy and feature importance estimation
XGBoost	Gradient boosting	Efficient modelling of complex nonlinear relationships

All models were trained using the same predictor variables and evaluated using identical validation procedures to ensure a consistent comparison of prediction performance^44,47,50^.

#### Feature importance analysis

Following model development, the relative importance of the predictor variables was evaluated using the impurity-based feature importance measure implemented in the Random Forest algorithm^50^. This analysis was performed to identify the pavement distress variables that contribute most strongly to PCI prediction and to support the engineering interpretation of pavement deterioration mechanisms.

## Forecasting and risk classification of pavement condition

Future pavement condition was estimated using the temporal evolution of the PCI observed during the four monitoring epochs. Because the measured PCI values exhibited an approximately linear deterioration trend for each roadway section throughout the monitoring period, a simple linear regression model was adopted to describe the relationship between PCI and time. The fitted regression equations were subsequently used to forecast PCI values for each roadway section up to the year 2030.The forecasting model can be expressed as in Eq. (12):

12 $$$$$$PC{I}_{t}={\beta}_{0}+{\beta}_{1}t$$$$$$

where $$$$$$PC{I}_{t}$$$$$$ is the predicted PCI at time *t*; $$$$$${\beta}_{0}$$$$$$ is the intercept; $$$$$${\beta}_{1}$$$$$$ is the annual rate of PCI change estimated from the observed data; *t* is the time expressed in years.

The regression coefficients were estimated using the observed PCI values from the four monitoring epochs. The resulting equations were then used to predict the future pavement condition of each roadway section assuming that the observed deterioration trend remains unchanged throughout the prediction period^39^.

The predicted PCI values were classified according to the standard PCI rating categories defined in ASTM D6433, including Excellent, Very Good, Good, Fair, Poor, Serious, and Failed as in Table 6. Based on these classifications, a pavement risk map was produced to identify roadway sections expected to experience significant deterioration before 2030. The resulting risk categories provide practical support for maintenance prioritization and proactive pavement management by identifying sections that require intervention before reaching critical condition levels (ASTM D6433-20).

**Table 6:** PCI risk classification used for forecasting.

Predicted PCI	Condition	Risk level	Recommended action
85–100	Excellent	Very Low	Routine monitoring
70–85	Very Good	Low	Preventive maintenance
55–70	Good	Moderate	Scheduled maintenance
40–55	Fair	High	Rehabilitation planning
25–40	Poor	Very High	Major rehabilitation
10–25	Serious	Critical	Immediate intervention
0–10	Failed	Extreme	Reconstruction

The range of the observed deterioration behaviour should be used to interpret the forecasting results. Extrapolation beyond the monitoring period assumes that future traffic loading, environmental conditions, and maintenance procedures stay equal to those observed during the study period.

## Results and discussion

This section presents the results obtained from the multi-temporal TLS observations conducted over the four observation epochs through 2022–2025. In order to determine the most significant parameters influencing the Pavement Quality Index (PCI), statistical analysis and machine-learning algorithms are used to investigate the correlations between individual distress kinds and pavement quality. In order to provide useful assistance for maintenance planning and decision-making, the forecasting models that have been built are utilized to estimate future pavement deterioration and categorize the roadway sections based on their anticipated risk levels.

### Roadway defects from TLS versus visual inspection

Conventional visual inspection and terrestrial laser scanning (TLS) were independently used to assess pavement surface distresses along the eight monitored roadway sections. A comparison of the measurements obtained using both approaches is presented in Table 7, which summarizes the capability of each method to detect and quantify the investigated pavement distress types.

**Table 7:** Comparison of defects resulted from visual inspection and TLS measurements for the studied roadway (all eight positions) July 2023.

Instrument position	Distress type	Measurement unit	Visual inspection results	From TLS observations
1	Longitudinal cracking	m	1.90	1.929
	Transverse cracking	m	2.25	2.287
	Bleeding	m^2^	Cannot measure	5.896
	Raveling	m^2^	Cannot measure	29.355
	Potholes	count/m^2^	3 potholes	3/0.46, 0.31, 0.73
2	Edge cracking	m	14.2	9.641
	Longitudinal cracking	m	12.1	14.662
	Slidage cracking	m^2^	Cannot measure	16.962
	Shoving	m	4.20	4.326
	Lane–shoulder drop-off	m	Cannot measure	10.790
	Polished aggregate	m^2^	288	288.82
	Raveling	m^2^	Cannot measure	28.33
	Potholes	count/m^2^	2 samples	2/0.36,0.51
	Rutting	mm	6	6.82
3	Block cracking	m^2^	2.73	2.735
	Longitudinal cracking	m	2.40	2.464
	Transverse cracking	m	2.50	2.532
	Raveling	m^2^	Cannot measure	12.835
	Potholes	count/m^2^	3 samples	3/ 0.21,0.37,0.27
	Edge cracking	m	12.6	12.615
4	Alligator cracking	m^2^	53.4	54.661
	Block cracking	m^2^	1.15	1.180
	Bleeding	m^2^	Cannot measure	82.280
	Longitudinal cracking	m	15.40	15.477
	Shoving	mm	7	7.845
5	Alligator cracking	m^2^	6.40	6.436
	Longitudinal cracking	m	4.10	4.140
	Raveling	m^2^	Cannot measure	6.536
	Potholes	count/m^2^	1 sample	1/ 0.36
	Patching	count/m^2^	1 sample	1/ 28.738
6	Alligator cracking	m^2^	60.70	60.737
	Edge cracking	m	7.20	7.251
	Raveling	m^2^	65.70	65.745
	Shoving	mm	10	10.1
7	Block cracking	m^2^	250	252.52
	Raveling	m^2^	230	234.68
8	Slidage cracking	m^2^	Cannot measure	80.777
	Shoving	mm	5.0	5.9
	Sags and pumps	m^2^	14	14.968
	Lane–shoulder drop-off	m	3.5	3.564
	Raveling	m^2^	15	15.964

As shown in Table 7, TLS measurements were generally consistent with those obtained through conventional visual inspection for pavement distresses that can be directly quantified during field surveys, including longitudinal cracking, transverse cracking, block cracking, alligator cracking, rutting, and shoving. The close agreement between the two methods confirms the capability of TLS to provide reliable quantitative measurements while maintaining consistency with conventional inspection practices. Table 7 also demonstrates a major advantage of TLS for pavement distresses that were recorded during the visual inspection as cannot be measured, including raveling, slippage cracking, polished aggregate, bleeding, and lane–shoulder drop-off. Unlike conventional visual surveys, TLS successfully quantified these defects by extracting their geometric characteristics directly from the three-dimensional point clouds. This capability extends pavement condition assessment beyond subjective visual observation and provides objective measurements for distress types that are difficult or impossible to quantify manually. The differences observed between the two methods were most evident for longitudinal and edge cracking at Position 2, where TLS measured greater crack lengths than those obtained through visual inspection. These differences are attributed to the ability of TLS to capture the complete three-dimensional geometry of pavement defects, whereas manual measurements may underestimate discontinuous or irregular crack patterns. Consequently, TLS reduces operator subjectivity and improves the repeatability and consistency of pavement distress measurements.

The findings of the present study are consistent with previous research demonstrating the advantages of TLS for pavement condition assessment. Barbarella et al.^3^ reported that TLS provides accurate and repeatable measurements of pavement surface characteristics while reducing the uncertainty associated with conventional visual inspection. Similarly^4^, showed that high-density TLS point clouds enable accurate detection and quantification of pavement distresses, including cracking, rutting, potholes, and surface deformation. The results obtained in this study further demonstrate that repeated TLS observations can provide a comprehensive quantitative database for monitoring pavement deterioration over time and support subsequent statistical analyses and machine-learning applications.

These findings demonstrate that TLS not only reproduces the measurements obtained through conventional visual inspection but also provides additional quantitative information for pavement distresses that cannot be reliably assessed using traditional field methods. The resulting high-resolution pavement distress database forms the basis for the deterioration analysis presented in the following sections.

### Multi-temporal roadway distress growth

**Fig. 6:**
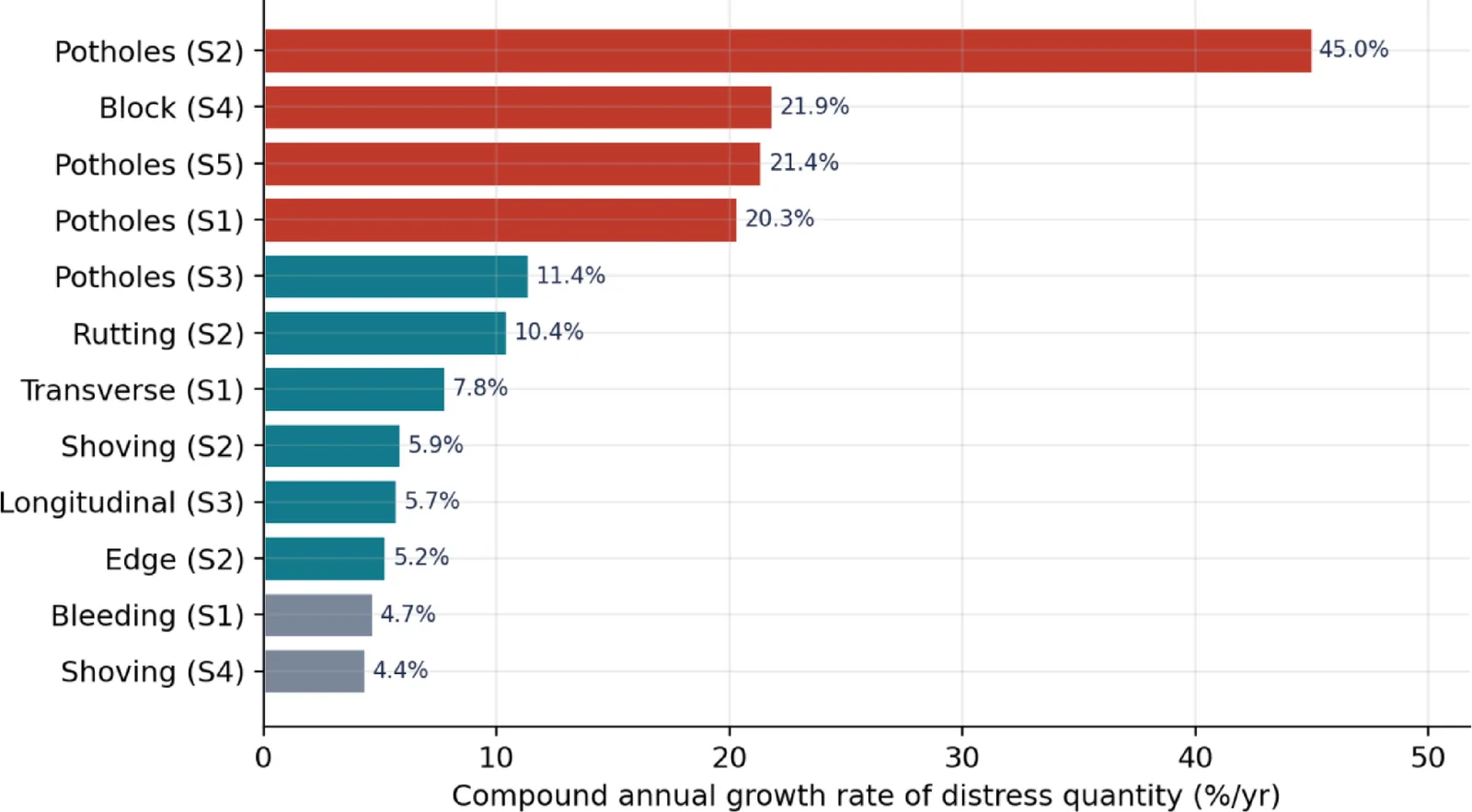
Compound annual growth rate of the fastest advancing distresses

The availability of four consecutive TLS surveys enabled the assessment of pavement distress evolution over time rather than relying on a single condition evaluation. The repeated measurements conducted between November 2022 and March 2025 provided a continuous record of distress progression, allowing deterioration rates to be quantified, rapidly developing distresses to be identified, and relatively stable pavement sections to be distinguished. This temporal assessment provides valuable information for understanding pavement deterioration mechanisms and supports the development of proactive maintenance strategies. The deterioration rates of the most rapidly increasing pavement distresses, calculated using the Compound Annual Growth Rate (CAGR), are presented in Fig. 6. Pothole development exhibited the highest deterioration rate among the investigated distresses. In Section “Study area, instruments and data acquisition”, pothole area increased from 0.71 to 1.69 m^2^ during the monitoring period, corresponding to an annual growth rate of approximately 45%. Similar increases were observed in Sections “Predictive modeling framework”, “Introduction”, and “Research methodology and plan”, where pothole growth exceeded 20% per year. Other distresses, including rutting, transverse cracking, and shoving, exhibited moderate growth rates ranging between 5 and 12% per year, whereas bleeding and several cracking types showed relatively limited progression.

The observed deterioration pattern indicates that different distress mechanisms do not contribute equally to pavement degradation. The rapid development of potholes reflects progressive localized surface deterioration and possible weakening of underlying pavement layers, making pothole growth an important early indicator of pavement performance decline. Although some cracking types exhibited lower annual growth rates, their continued progression may contribute to water infiltration and accelerate subsequent structural deterioration. Therefore, monitoring distress progression provides additional information beyond the assessment of current distress severity alone. The complete temporal measurements obtained from TLS for all roadway sections and observation epochs are summarized in Table 8. The repeated measurements demonstrate the capability of TLS to generate a quantitative deterioration history for each pavement section. Among the investigated sections, Section “Study area, instruments and data acquisition” showed the most pronounced deterioration, characterized by continuous increases in longitudinal cracking, edge cracking, rutting, lane–shoulder drop-off, and pothole development. In contrast, Sections “Forecasting and risk classification of pavement condition” and “Decision-support framework for pavement maintenance management” exhibited relatively limited changes during the monitoring period, indicating comparatively stable pavement conditions.

**Table 8:** Complete surface roadway defects measurements from TLS observations for all sections and all observations epochs.

Instrument position	Distress type (unit)	November 2022	July 2023	April 2024	March 2025
1	Longitudinal cracking (m)	1.871	1.929	1.941	1.965
	Transverse cracking (m)	2.187	2.287	2.315	2.606
	Bleeding (m^2^)	5.312	5.896	5.905	5.914
	Raveling (m^2^)	29.141	29.355	29.7	30.4
	Potholes (m^2^)	3/0.46,0.31,0.73	3/0.46,0.31,0.73	3/0.46,0.31,0.73	4/0.46,0.31,0.73,0.81
2	Edge cracking (m)	9.421	9.641	10.233	10.614
	Longitudinal cracking (m)	14.199	14.662	15.208	15.446
	Slidage cracking (m^2^)	16.285	16.962	17.230	17.405
	Shoving (mm)	4.170	4.326	4.511	4.764
	Lane–shoulder drop-off (m)	10.482	10.790	10.922	11.234
	Polished aggregate (m^2^)	287.8	288.82	289.32	289.84
	Raveling (m^2^)	27.82	28.33	28.611	28.724
	Potholes (m^2^)	2: 0.26,0.45	2: 0.36,0.51	2: 0.46,0.74	3: 0.46,0.78,0.45
	Rutting (mm)	6.22	6.82	7.21	7.84
3	Block cracking (m^2^)	2.63	2.735	2.738	2.88
	Longitudinal cracking (m)	2.33	2.464	2.52	2.652
	Transverse cracking (m)	2.50	2.532	2.544	2.611
	Raveling (m^2^)	12.97	12.835	12.97	13.22
	Potholes (m^2^)	3: 0.21,0.37,0.19	3: 0.21,0.37,0.27	3: 0.21,0.39,0.31	4: 0.21,0.37,0.19,0.22
	Edge cracking (m)	12.405	12.615	12.92	12.951
4	Alligator cracking (m^2^)	54.245	54.661	54.682	55.055
	Block cracking (m^2^)	1.05	1.180	1.651	1.665
	Bleeding (m^2^)	81.6	82.280	83.12	83.22
	Longitudinal cracking (m)	15.30	15.477	15.48	15.49
	Shoving (mm)	7.11	7.845	7.86	7.86
5	Alligator cracking (m^2^)	6.402	6.436	6.511	6.672
	Longitudinal cracking (m)	4.055	4.140	4.140	4.461
	Raveling (m^2^)	6.4	6.536	6.554	6.558
	Potholes (m^2^)	1: 0.28	1: 0.36	1: 0.36	1: 0.44
	Patching (m^2^)	28.738	28.738	28.828	29.122
6	Alligator cracking (m^2^)	60.70	60.737	60.822	60.823
	Edge cracking (m)	7.205	7.251	7.266	7.331
	Raveling (m^2^)	65.711	65.745	65.811	65.811
	Shoving (mm)	9.9	10.1	10.1	10.3
7	Block cracking (m^2^)	252.3	252.52	253.14	253.66
	Raveling (m^2^)	232.44	234.68	235.69	238.44
8	Slidage cracking (m^2^)	80.633	80.777	80.787	80.911
	Shoving (mm)	5.6	5.9	5.9	6.1
	Sags and pumps (m^2^)	14.244	14.968	15.22	15.28
	Lane–shoulder drop-off (m)	3.511	3.564	3.566	3.606
	Raveling (m^2^)	15.66	15.964	15.986	15.987

The different deterioration rates observed among pavement sections highlight the influence of local traffic loading, pavement structure, and environmental exposure on distress progression. Sections experiencing rapid growth in potholes, rutting, and surface deformation are more likely to require earlier maintenance intervention compared with sections dominated by slowly developing distresses. This finding agrees with previous pavement deterioration studies, which reported that localized surface failures such as potholes and rutting are strongly associated with accelerated pavement performance decline because they reflect the combined effects of traffic loading, moisture infiltration, and progressive material degradation^51^.

The results are also consistent with previous applications of laser-based pavement monitoring, where repeated three-dimensional surface measurements were shown to provide reliable information for tracking pavement deterioration processes. Beshr et al.^52^ demonstrated that repeated TLS observations enable accurate detection of pavement surface changes that may not be captured through conventional inspection methods. Similarly,^4^ emphasized that high-resolution point-cloud measurements allow detailed quantification of pavement distress characteristics and provide a reliable basis for deterioration analysis.

The multi-temporal TLS observations in this study therefore provide more than a record of distress magnitude; they enable identification of deterioration trends and prioritization of critical roadway sections. By integrating distress growth rates with subsequent PCI analysis and predictive modelling, TLS-based monitoring provides a practical framework for supporting condition-based maintenance planning and reducing the risk of unexpected pavement failure.

### PCI deterioration for roadway sections

**Fig. 7:**
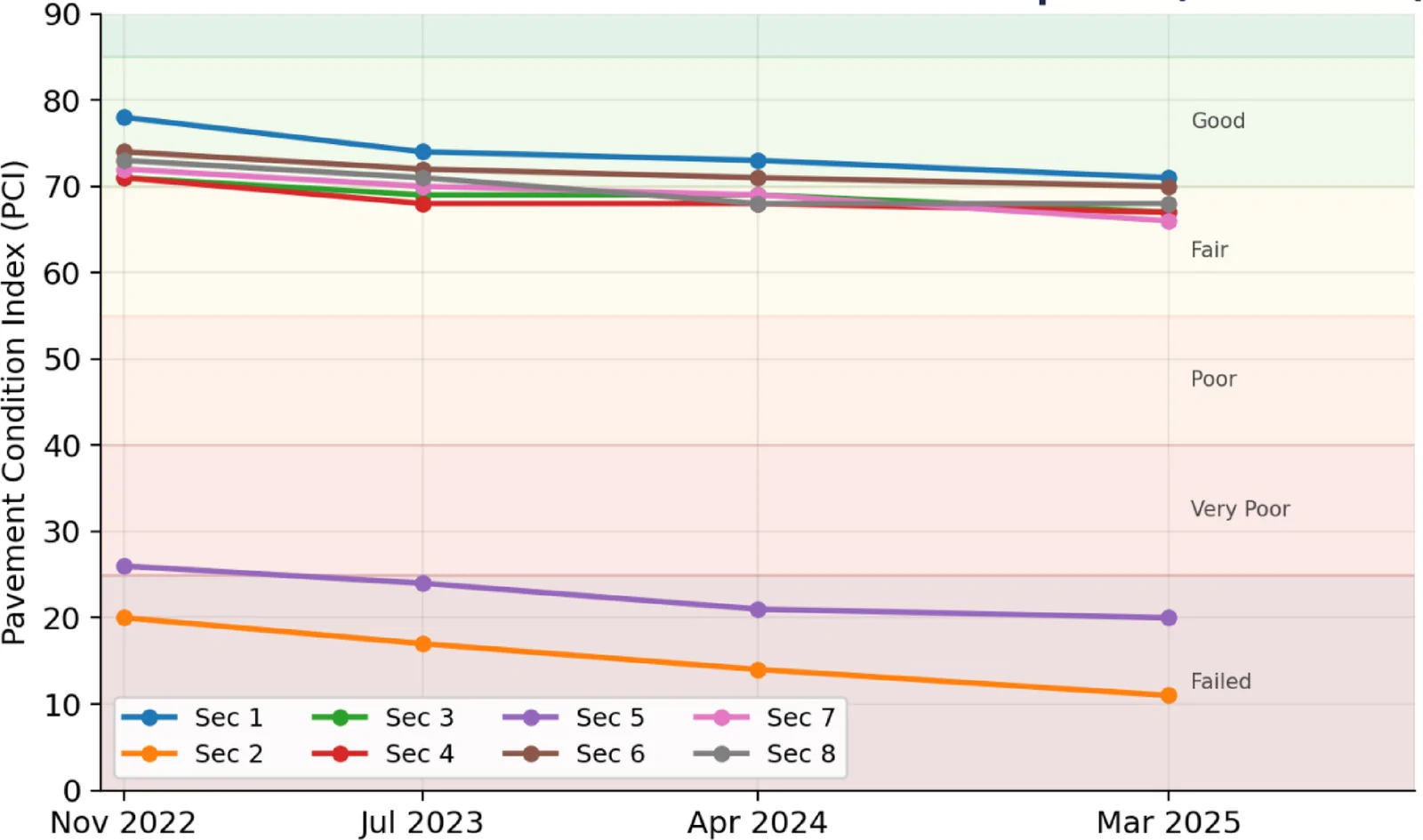
Measured PCI of all eight sections for all four observations epochs with risk bands

The temporal evolution of the Pavement Condition Index (PCI) provides an overall assessment of pavement performance along the study corridor during the four monitoring epochs. The measured PCI values obtained from the PAVER system, together with the corresponding condition loss and annual deterioration rates, are summarized in Tables 9 and 10, while the variation of PCI over time for the eight roadway sections is illustrated in Fig. 7.

**Table 9:** Average PCI values from TLS observations (using PAVER program) for all sections and all observations epochs.

Section (position)	November 2022	July 2023	April 2024	March 2025
1	78	74	73	71
2	20	17	14	11
3	71	69	69	67
4	71	68	68	67
5	26	24	21	20
6	74	72	71	70
7	72	70	69	66
8	73	71	68	68

**Table 10:** PCI deterioration and annual loss rate for all roadway sections.

Section	PCI 2022	PCI 2025	Loss (pts)	Loss (%)	Rate (pts/yr)
1	78	71	7	9.0	− 2.79
2	20	11	9	45.0	− 3.85
3	71	67	4	5.6	− 1.55
4	71	67	4	5.6	− 1.50
5	26	20	6	23.1	− 2.67
6	74	70	4	5.4	− 1.65
7	72	66	6	8.3	− 2.46
8	73	68	5	6.8	− 2.26

As presented in Table 9, all investigated roadway sections experienced a continuous reduction in PCI during the monitoring period, confirming an overall deterioration trend throughout the study corridor. However, the magnitude of deterioration varied considerably among sections. Sections “Introduction”, “Research methodology and plan”, “Integrated data processing and analysis”, “Forecasting and risk classification of pavement condition”, “Results and discussion”, and “Decision-support framework for pavement maintenance management” maintained relatively higher PCI values, decreasing gradually from the Good condition category toward the Fair category. In contrast, Sections “Study area, instruments and data acquisition” and “Predictive modeling framework” exhibited substantially lower initial PCI values and continued deterioration, indicating poorer pavement performance compared with the remaining sections.

**Fig. 8:**
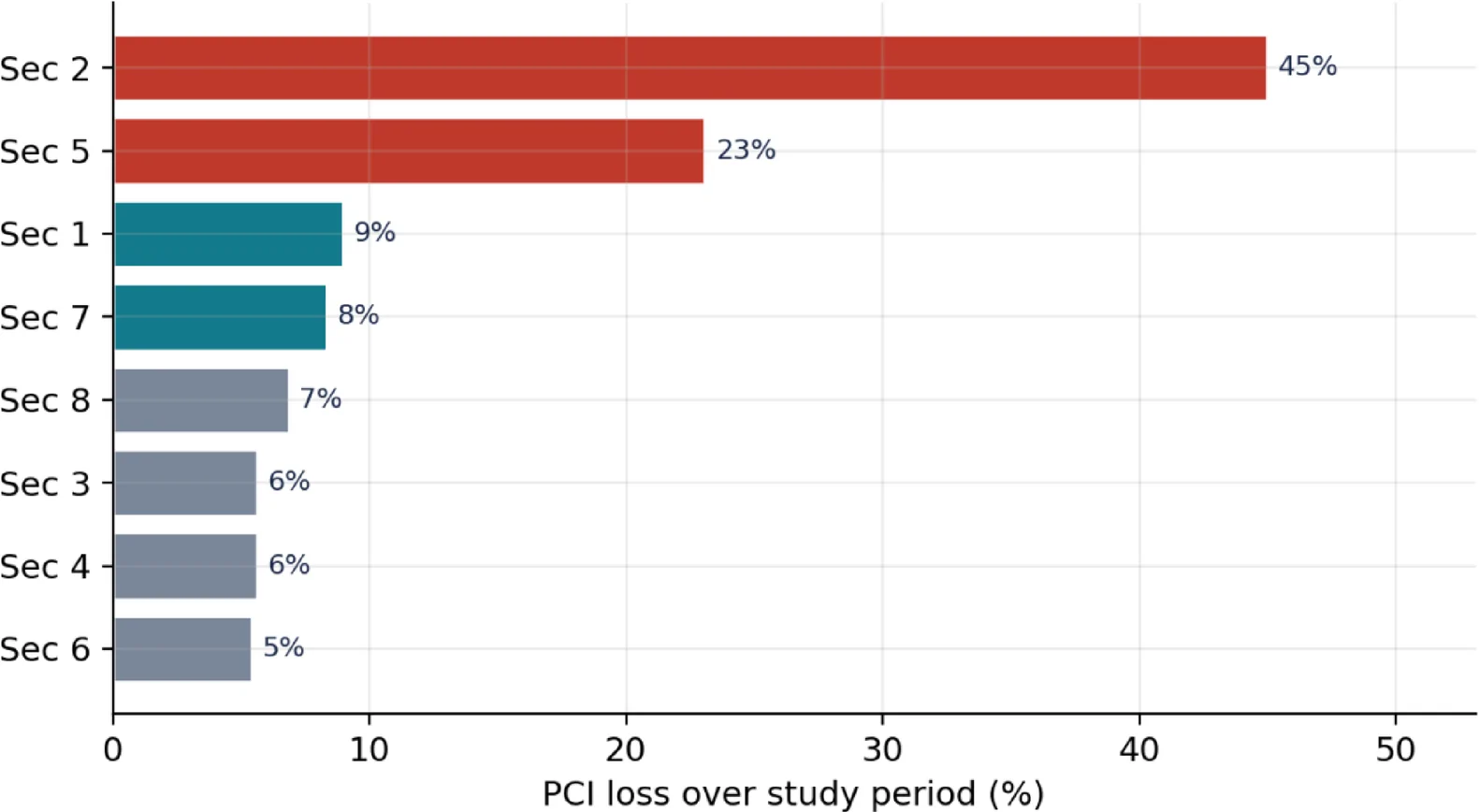
Total PCI loss for all roadway sections over the study period (2022–2025)

As shown in Fig. 7, the PCI trajectories can be divided into two distinct groups. The first group, consisting of Sections “Introduction”, “Research methodology and plan”, “Integrated data processing and analysis”, “Forecasting and risk classification of pavement condition”, “Results and discussion”, and “Decision-support framework for pavement maintenance management”, exhibited relatively consistent and gradual deterioration patterns, with nearly parallel PCI trends throughout the observation period. This behavior indicates a relatively stable deterioration process and supports the application of linear trend modelling for future PCI prediction. The second group, represented by Sections “Study area, instruments and data acquisition” and “Predictive modeling framework”, showed considerably lower PCI values and faster deterioration rates. The increasing difference between these two groups indicates that pavement deterioration was not uniform across the corridor, resulting in increasing variability in network condition over time. The magnitude of PCI reduction for each section is further illustrated in Fig. 8, while the corresponding deterioration rates are summarized in Table 10. Section “Study area, instruments and data acquisition” experienced the highest condition loss, decreasing from PCI 20 in November 2022 to PCI 11 in March 2025, corresponding to a 45% reduction over the monitoring period. Section “Predictive modeling framework” also showed considerable deterioration, with a 23.1% PCI reduction. In comparison, the remaining sections experienced relatively limited losses ranging from 5.4 to 9.0%.

The observed differences in PCI deterioration rates demonstrate that pavement sections within the same roadway corridor may exhibit substantially different performance behaviors due to variations in traffic loading, pavement structure, drainage conditions, and existing distress severity. Similar observations have been reported in pavement management studies, where heterogeneous deterioration patterns were attributed to differences in environmental exposure, loading conditions, and initial pavement condition^12,53^. The faster deterioration observed in critical sections is consistent with the concept that pavements with lower initial condition levels generally experience accelerated performance decline due to the interaction between existing damage and increasing structural vulnerability. The results also support previous findings that PCI-based condition monitoring can provide an effective framework for maintenance prioritization by identifying sections requiring early intervention^54^. In the present study, Sections “Study area, instruments and data acquisition” and “Predictive modeling framework” accounted for the majority of the total condition loss despite representing only a limited portion of the investigated corridor. Therefore, prioritizing these rapidly deteriorating sections would provide the greatest benefit in maintaining overall network condition and preventing more extensive rehabilitation requirements.

### Pearson correlation analysis between distress and PCI

**Fig. 9:**
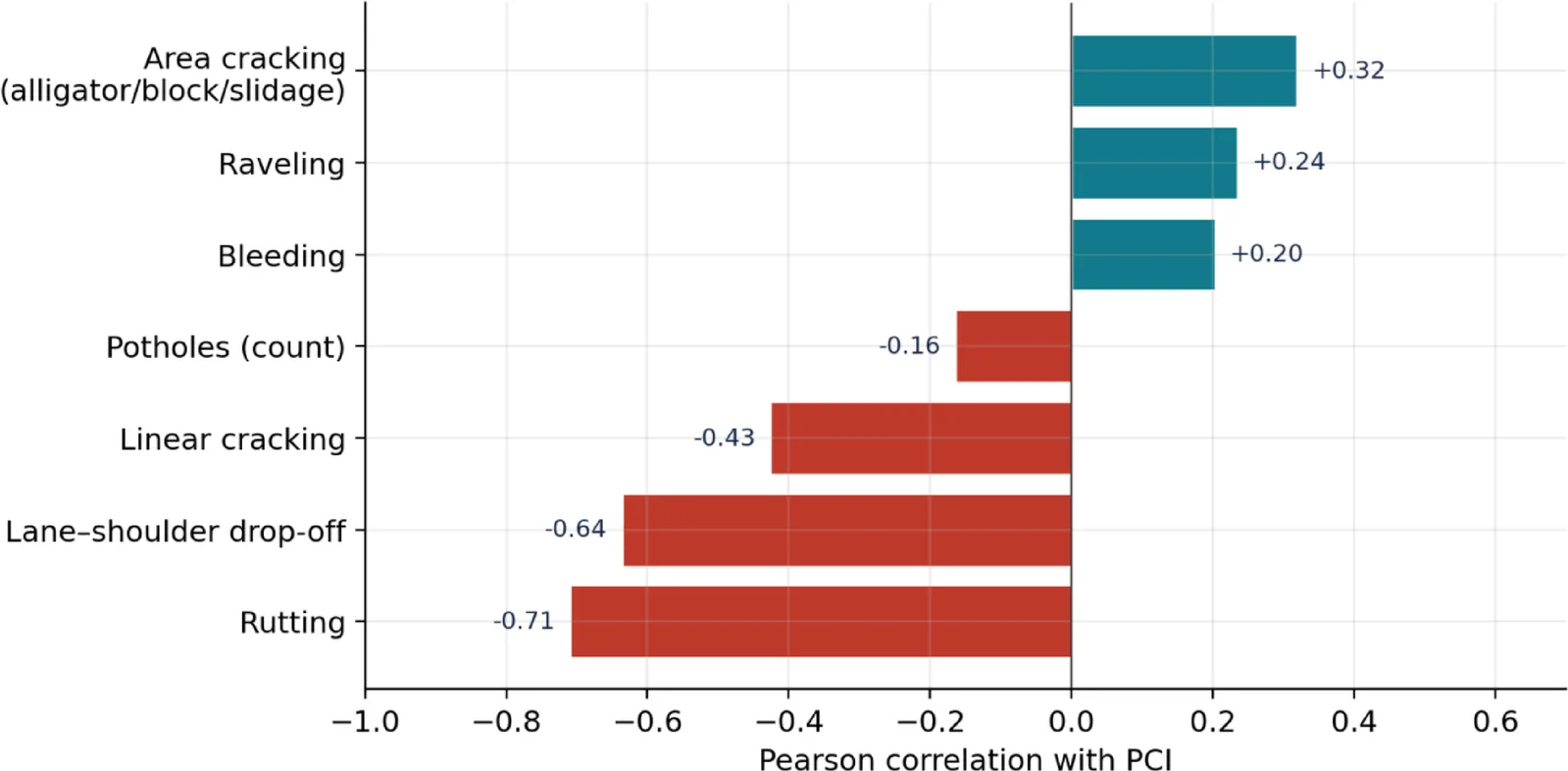
Pearson correlation between distress type and PCI (32 sections for all four observations epochs)

Pearson correlation analysis was performed to evaluate the relationship between individual pavement distress variables and the Pavement Condition Index (PCI) using the 32 section–epoch observations. The correlation coefficients and corresponding significance levels are summarized in Table 11, while the graphical representation of the relationships is presented in Fig. 9.

**Table 11:** Pearson correlation between each distress and PCI (computed from the 32 section–epoch observations).

Distress/index	Correlation with PCI (r)	*p*-value
Rutting	− 0.71	< 0.001
Lane–shoulder drop-off	− 0.64	< 0.001
Linear cracking	− 0.43	0.015
Composite distress index	− 0.40	0.023
Potholes (count)	− 0.16	0.369
Bleeding	+ 0.20	0.263
Raveling	+ 0.24	0.195
Area cracking (alligator/block/slidage)	+ 0.32	0.074

As shown in Table 11, rutting exhibited the strongest negative correlation with PCI (r =  − 0.71, *p* < 0.001), followed by lane–shoulder drop-off (r =  − 0.64, *p* < 0.001) and linear cracking (r =  − 0.43, *p* = 0.015). The composite distress index also showed a significant negative relationship with PCI (r =  − 0.40, *p* = 0.023), indicating that the overall increase in pavement distress was associated with reduced pavement condition.

The strong negative relationship observed for rutting and lane–shoulder drop-off indicates that these distresses are closely associated with pavement serviceability reduction. However, these correlations are partly influenced by their concentration in the most deteriorated roadway sections, particularly Sections “Study area, instruments and data acquisition” and “Predictive modeling framework”. Therefore, the strong statistical relationship reflects both the direct influence of these distresses and their association with sections exhibiting overall poor pavement condition. In comparison, linear cracking showed a moderate negative correlation with PCI despite being distributed across a larger proportion of the study corridor, suggesting that it provides a more representative indicator of general pavement deterioration.

In contrast, potholes, bleeding, raveling, and area-based cracking showed relatively weak relationships with PCI. These results indicate that distress magnitude alone does not always represent the overall pavement condition, as the influence of a distress depends on its severity, location, and structural significance. For example, Section “Results and discussion” contained considerable areas of block cracking and raveling but maintained a higher PCI compared with Sections “Study area, instruments and data acquisition” and “Predictive modeling framework”, where more critical distresses such as rutting and pothole development were dominant. Therefore, pavement condition assessment should consider not only distress quantity but also distress type and its contribution to pavement functional performance.

The observed correlation behavior agrees with previous pavement management studies showing that structural-related distresses, particularly rutting and cracking, generally exhibit stronger relationships with pavement condition indicators than individual surface defects^12,55^ Rutting is often considered a critical indicator of pavement structural deterioration because it reflects permanent deformation caused by repeated traffic loading, while cracking progression can accelerate deterioration through moisture infiltration and weakening of pavement layers^55^. However, the relatively weak correlation of some distress types observed in this study highlights the importance of interpreting individual distress measurements within their pavement context rather than relying solely on statistical association.

### Multiple regression model and its adequacy

Following the correlation analysis, a multiple linear regression model was developed to evaluate the combined influence of pavement distress variables on PCI. Unlike Pearson correlation, which evaluates the relationship between individual distress variables and PCI independently, the regression model estimates the contribution of each predictor while considering the simultaneous effects of the remaining variables. The fitted regression coefficients were evaluated using the coefficient of determination R^2^, adjusted R^2^, F-test, residual analysis, and individual predictor significance tests. Applying the least-squares estimator described in Eq. (13) to the 32 section–epoch observations resulted in the following fitted PCI prediction equation:

13 $$$$$$ \begin{aligned} PCI & = 25.84 + 1.91 \cdot Linear - 0.87 \cdot AreaCrack \\ & \quad + 1.14 \cdot Ravel + 0.76 \cdot Bleed - 0.68 \cdot Potholes \\ & \quad - 50.78 \cdot Rut + 26.20 \cdot Dropoff \\ \end{aligned} $$$$$$

The developed regression model showed a statistically significant relationship between pavement distress variables and PCI, with an F-value of 17.15 (7 and 24 degrees of freedom) and a significance level of *p* ≈ 6 × 10^−8^. The model explained a substantial proportion of the observed PCI variability, achieving an R^2^ value of 0.833 and an adjusted R^2^ value of 0.785. Furthermore, the residuals showed an approximately normal distribution (Shapiro–Wilk test, *p* = 0.41), with a regression standard error of approximately 9.3 PCI points, indicating that the.

As presented in Table 12, six of the seven investigated predictors showed statistically significant relationships with PCI. Rutting exhibited the strongest contribution to PCI reduction (t = − 6.49, *p* < 0.001), followed by lane–shoulder drop-off (t = 5.56, *p* < 0.001), raveling (t = 4.63, *p* < 0.001), bleeding (t = 4.50t, *p* < 0.001), area cracking (t =  − 3.90, *p* < 0.001), and linear cracking (t = 3.66, *p* = 0.001). In contrast, pothole count was not statistically significant (*p* = 0.793), indicating that its contribution could not be independently distinguished within the fitted regression framework. The large negative coefficient associated with rutting (− 50.78) indicates its strong influence on PCI reduction and reflects the high severity assigned to permanent deformation in pavement condition evaluation. However, some regression coefficients exhibited unexpected signs, such as the positive coefficients associated with lane–shoulder drop-off and raveling. These results are attributed to multicollinearity among pavement distress variables, where several distresses occur simultaneously within the same deteriorated roadway sections. Under such conditions, OLS regression may have difficulty separating the individual contribution of correlated predictors, causing changes in coefficient magnitude and direction despite maintaining good overall predictive performance. Therefore, the regression coefficients should be interpreted as components of a combined statistical model rather than independent causal effects. This interpretation is consistent with the correlation analysis presented in Section “6.4” and is further supported by the machine-learning feature importance analysis presented later in Section “6.6”, where the relative contribution of distress variables is evaluated using a non-linear modelling framework. Similar limitations of regression-based pavement deterioration models have been reported in previous studies, particularly when predictor variables are strongly correlated or when the available dataset is relatively limited^56^. The suitability of the linear regression model was further assessed by comparing model complexity and explanatory capability. Although the inclusion of quadratic terms increased the in-sample adjusted R^2^ value and reduced the Akaike Information Criterion (AIC), the resulting model required a substantially larger number of predictors relative to the available observations. Given that the dataset consisted of 32 observations representing only eight roadway sections, increasing model complexity may lead to overfitting and reduced generalization capability. Therefore, following the principle of model parsimony, the linear model was retained as the most appropriate explanatory framework because it provides a balance between statistical adequacy, interpretability, and applicability for pavement management decisions^39,57^. The regression model therefore provides an interpretable description of the relationship between TLS-derived pavement distress measurements and PCI, while the temporal deterioration model and machine-learning approaches provide complementary perspectives for forecasting and predictive analysis.

**Table 12:** OLS regression coefficients for PCI with standard errors, t-statistics and significance (Where: *** *p* < 0.001, ** *p* < 0.01, * *p* < 0.05, n.s. = not significant).

Predictor	Coefficient β̂	Std. error	t	*p*-value	Significance
Intercept	25.837	6.398	4.04	< 0.001	***
Linear cracking	1.910	0.522	3.66	0.001	**
Area cracking	− 0.872	0.224	− 3.90	< 0.001	***
Raveling	1.135	0.245	4.63	< 0.001	***
Bleeding	0.755	0.168	4.50	< 0.001	***
Potholes	− 0.684	2.583	− 0.26	0.793	n.s
Rutting	− 50.780	7.828	− 6.49	< 0.001	***
Lane–shoulder drop-off	26.199	4.711	5.56	< 0.001	***

### Machine learning comparison and feature importance

**Fig. 10:**
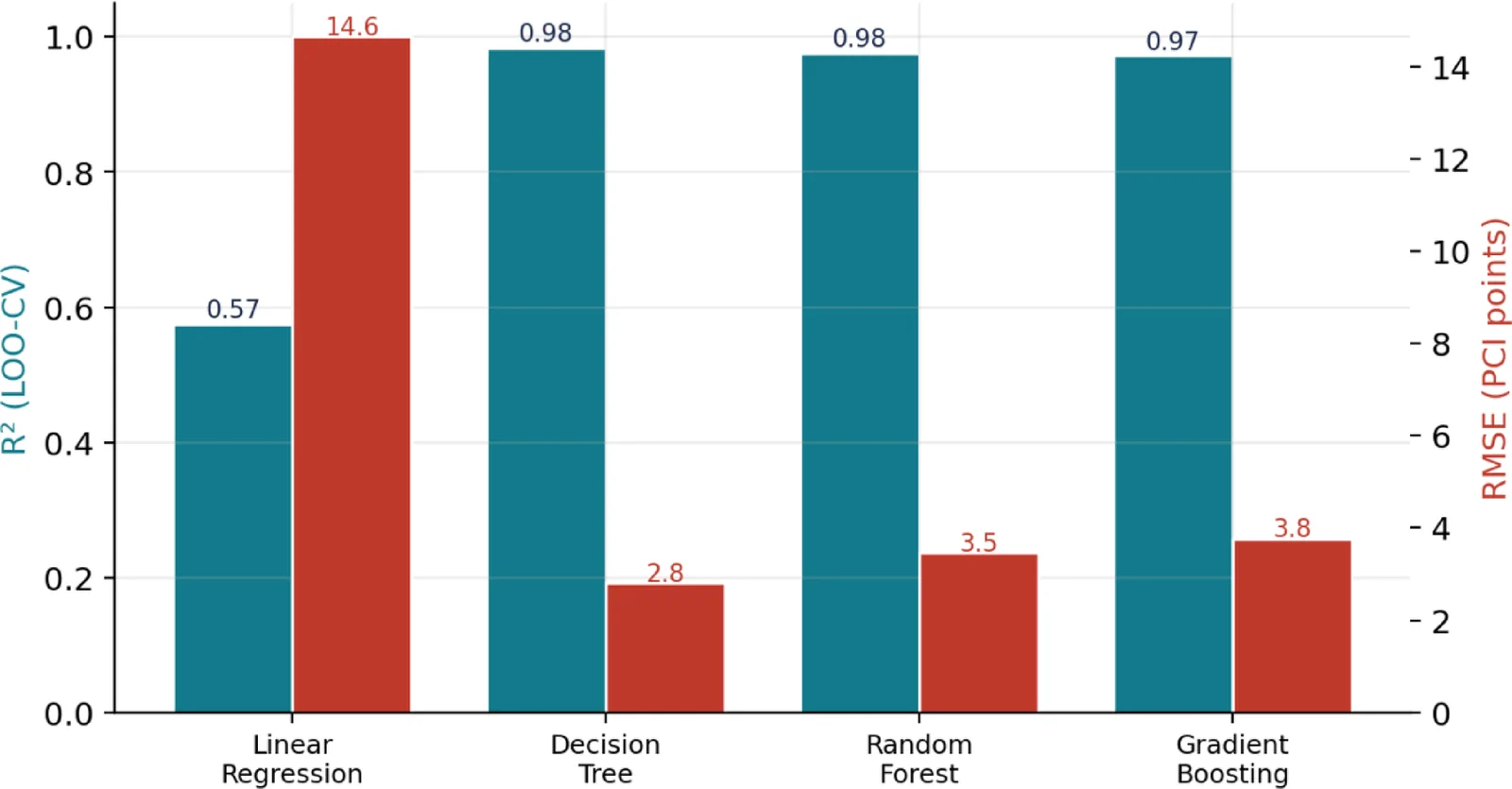
Model accuracy (R^2^ and RMSE) under leave-one-out cross-validation

Although the multiple regression model provided valuable information regarding the relationship between pavement distress variables and PCI, its ability to represent complex nonlinear interactions between distress characteristics is limited. Therefore, four supervised machine-learning algorithms were evaluated to examine their capability for predicting PCI from the TLS-derived distress variables. In addition to evaluating prediction accuracy, feature importance analysis was conducted to identify the distress variables contributing most significantly to PCI variation. The predictive performance of the evaluated models under the leave-one-out cross-validation (LOO-CV) framework is presented in Table 13, while the comparison of model accuracy is illustrated in Fig. 10.

**Table 13:** Predictive model performance (in-corridor leave-one-out cross-validation).

Model	R^2^ (LOO-CV)	RMSE (PCI pts)
Linear regression	0.575	14.64
Decision tree	0.984	2.8
Random forest	0.976	3.46
Gradient boosting	0.972	3.77

**Fig. 11:**
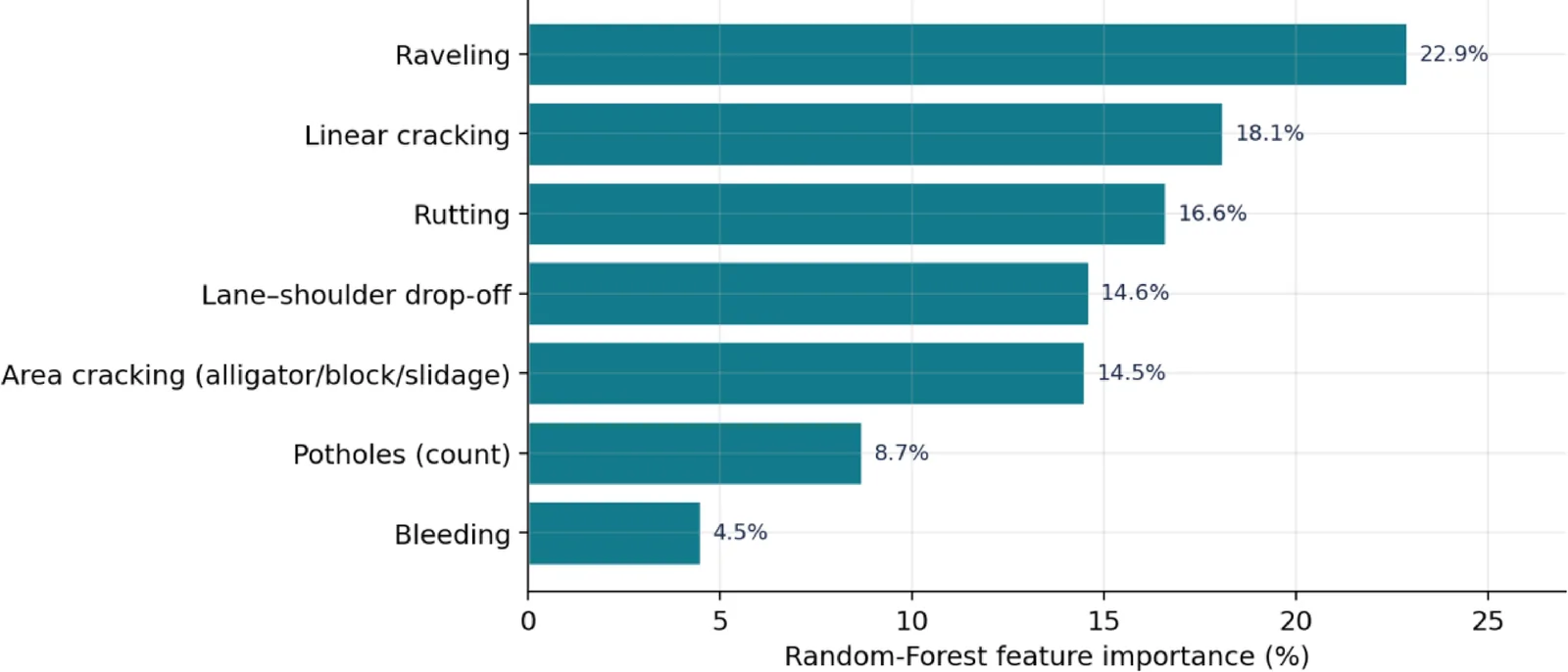
Random-Forest feature importance for PCI prediction

As shown in Table 13, the tree-based models achieved substantially higher predictive accuracy than the linear regression model under the LOO-CV framework. The Decision Tree, Random Forest, and Gradient Boosting models achieved R^2^ values ranging from 0.972 to 0.984, with RMSE values below 4 PCI points, whereas the linear regression model achieved a lower R^2^ value of 0.575 and a higher RMSE of 14.64 PCI points. These results indicate that nonlinear learning algorithms were better able to represent the complex relationships between pavement distress characteristics and PCI. The improved performance of the ensemble learning approaches is consistent with previous pavement condition prediction studies, where Random Forest and boosting-based algorithms demonstrated strong capability in capturing nonlinear relationships among pavement distress variables^47,48^. However, the high LOO-CV accuracy should be interpreted carefully because the validation samples originated from the same roadway sections included in the training dataset at different observation epochs. The relative contribution of each distress variable was evaluated using the Random Forest feature importance measure. The resulting importance ranking is presented in Table 14 and visualized in Fig. 11.

**Table 14:** Random-Forest feature importance for PCI prediction (computed).

Variable	Random-Forest importance (%)
Raveling	22.9
Linear cracking	18.1
Rutting	16.6
Lane–shoulder drop-off	14.6
Area cracking (alligator/block/slidage)	14.5
Potholes (count)	8.7
Bleeding	4.5

The feature importance analysis identified raveling (22.9%), linear cracking (18.1%), rutting (16.6%), and lane–shoulder drop-off (14.6%) as the most influential predictors of PCI variation. The dominance of raveling and linear cracking, which were distributed across multiple roadway sections, complements the Pearson correlation analysis, where rutting and lane–shoulder drop-off showed the strongest direct correlations with PCI due to their concentration in the most deteriorated sections. This difference highlights the advantage of machine-learning methods in evaluating the combined contribution of multiple variables rather than relying only on individual statistical relationships. The generalization capability of the developed models was further examined using the more rigorous leave-one-section-out (LOSO) validation approach. Under this framework, all models showed substantially reduced performance, with negative R^2^ values and RMSE values approaching 30 PCI points. This reduction indicates that the models were unable to reliably predict the condition of an entirely unseen roadway section based only on distress characteristics. The difference between the LOO-CV and LOSO results demonstrates the importance of considering the structure and independence of pavement datasets during machine-learning evaluation. The high LOO-CV performance reflects the availability of repeated observations from the same roadway sections, allowing the models to learn section-specific distress patterns. In contrast, the LOSO validation represents a more realistic assessment of transferability to new roadway sections, where the available dataset size is insufficient for robust cross-road prediction. Therefore, the machine-learning models developed in this study should be interpreted primarily as tools for identifying influential distress variables and describing relationships within the investigated corridor rather than as universally transferable prediction models. The feature importance results provide valuable insight into the dominant deterioration mechanisms, while future applications involving larger and more diverse roadway datasets may enable the development of more generalizable predictive models.

### Forecast PCI values for roadways sections at year 2030

The future pavement condition of the study roadway was evaluated using the PCI values obtained from the four TLS observation epochs. For each roadway section, a temporal deterioration model was developed by fitting PCI as a function of time, and the resulting models were used to estimate future PCI values up to the year 2030. The predicted PCI values were then classified according to the standard PCI condition categories to identify sections requiring future maintenance intervention.

The temporal PCI models showed strong agreement with the observed data, with an average coefficient of determination (R^2^) of 0.91 and values ranging from 0.76 to 0.995 among the eight roadway sections. These results indicate that the observed deterioration trends during the monitoring period can be reasonably represented using linear models for short-term forecasting. Therefore, the temporal prediction approach was considered more appropriate for this dataset than distress-based prediction models, which showed limited generalization capability under the LOSO validation framework The forecast PCI values and corresponding risk classifications for 2030 are summarized in Table 15**.**

**Table 15:** Forecasting PCI values and risk classification for roadway sections at year 2030.

Roadway section	PCI at year 2025	PCI at year 2030 (forecasting values)	Risk class at year 2030
1	71	55	Fair
2	11	0	Failed
3	67	58	Fair
4	67	58	Fair
5	20	4	Failed
6	70	60	Fair
7	66	52	Poor
8	68	54	Poor

As presented in Table 15, substantial differences in future pavement condition are expected among the investigated roadway sections. Section “Study area, instruments and data acquisition” is predicted to reach a PCI value close to zero, while Section “Predictive modeling framework” is expected to decrease to approximately PCI 4 by 2030. Both sections are therefore projected to reach the Failed condition category, indicating a high priority for rehabilitation intervention. In comparison, Sections “Introduction”, “Research methodology and plan”, “Integrated data processing and analysis”, and “Forecasting and risk classification of pavement condition” are predicted to remain within the Fair condition category, whereas Sections “Results and discussion” and “Decision-support framework for pavement maintenance management” are expected to approach the Poor condition category.

**Fig. 12:**
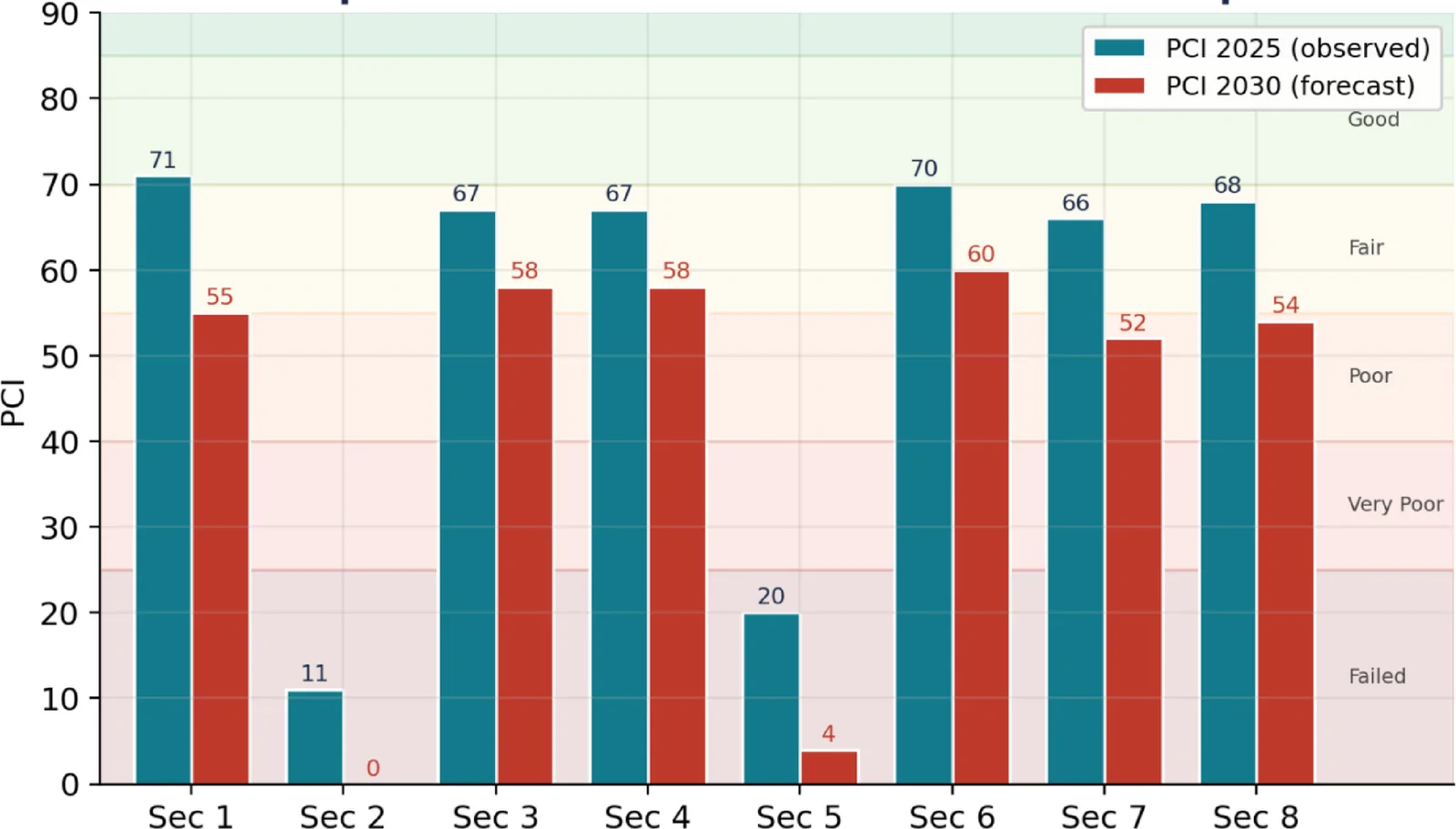
Observed values (2025) versus forecasting values (2030) of PCI by roadway sections

As illustrated in Fig. 12, the forecast results reveal two distinct deterioration patterns within the study corridor. The first group, consisting of Sections “Study area, instruments and data acquisition” and “Predictive modeling framework”, exhibits accelerated deterioration and reaches critical condition levels by 2030. The second group includes the remaining sections, which experience gradual PCI reduction while maintaining relatively serviceable conditions. This difference demonstrates that pavement deterioration is not uniform across the roadway network and that maintenance priorities should focus on sections exhibiting rapid condition decline. The observed forecasting behaviour agrees with previous pavement management studies showing that deterioration models based on historical pavement condition data can provide valuable support for maintenance planning and intervention prioritization^8,51^. However, the reliability of long-term forecasts depends strongly on the availability of sufficient historical observations and the assumption that future traffic loading, environmental conditions, and maintenance activities remain comparable to those during the calibration period. In this study, the forecasting approach assumes that the current deterioration rates will continue and that no major rehabilitation activities will be performed before 2030. Although the linear models provide a practical short-term forecasting framework, pavement deterioration may become nonlinear as sections approach failure conditions. Therefore, future updates incorporating additional TLS monitoring epochs and more advanced deterioration models, such as nonlinear regression or Markov-based approaches, may further improve long-term prediction accuracy.

### Risk classification

**Fig. 13:**
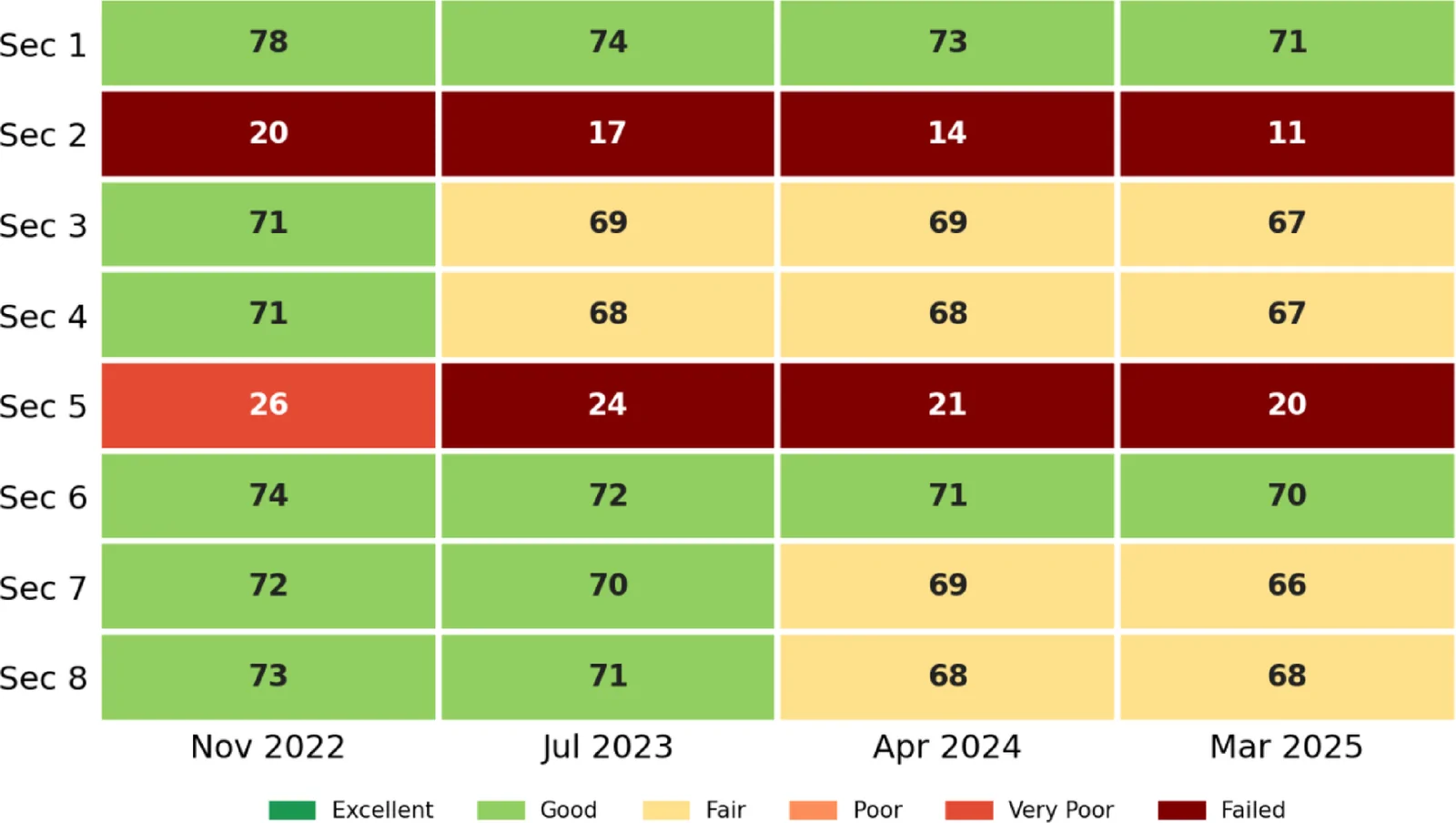
Risk classification of all sections across the four epochs

The PCI-based risk classification was developed to visualize the spatial and temporal evolution of pavement condition throughout the monitoring period. Figure 13 presents the risk classification of all roadway sections across the four observation epochs, allowing the progression of pavement condition categories and the identification of sections requiring different levels of intervention.

As shown in Fig. 13, the investigated roadway sections exhibited two distinct deterioration patterns. Section “Study area, instruments and data acquisition” remained within the Failed condition category throughout the monitoring period, while Section “Predictive modeling framework” experienced a transition from the Very Poor category to the Failed category during the observation period. In contrast, the remaining six sections showed gradual movement from Good Toward Fair condition categories, indicating slower deterioration and a greater potential for preservation-based maintenance strategies. The risk classification provides a clear representation of both the current pavement condition and the rate of deterioration. A horizontal interpretation of the classification matrix illustrates the progression of individual sections over time, whereas the latest observation epoch (March 2025) provides an overview of the current network condition. At the end of the monitoring period, two sections were classified as Failed, while the remaining sections were located mainly within the Good–Fair condition range, indicating different maintenance priorities across the roadway corridor. The identification of these two deterioration groups has important implications for pavement management. Sections exhibiting critical deterioration require immediate rehabilitation planning to prevent further structural degradation, whereas sections with moderate deterioration may benefit from preventive maintenance treatments to extend their service life. This risk-based approach enables maintenance resources to be allocated according to actual pavement needs rather than relying only on periodic inspection schedules. The integration of multi-temporal TLS observations with PCI-based risk classification provides an objective and practical decision-support framework for pavement asset management. Similar approaches have been recommended in pavement management studies, where condition-based classification systems are used to prioritize maintenance activities and optimize the allocation of limited rehabilitation resources^12,58^. In this study, the risk classification further demonstrates the value of combining high-resolution distress measurements with temporal pavement performance analysis to support proactive maintenance planning.

## Decision-support framework for pavement maintenance management

The results obtained from TLS-based distress quantification, PCI deterioration analysis, risk classification and 2030 condition forecasting provide a basis for developing a condition based maintenance and rehabilitation strategy for the investigated roadway surface. The aim of this strategy is to maximize pavement service life and optimize long-term maintenance resources by assigning appropriate interventions according to the current pavement condition and dominant distress mechanisms. The proposed approach follows the principle that maintenance treatments should be matched to the PCI condition category and the prevailing pavement distress type. Early intervention before pavement conditions reach critical deterioration levels can reduce the need for extensive rehabilitation and improve the cost-effectiveness of pavement management decisions. Based on the PCI ranges and observed distress characteristics within the study corridor, a maintenance and rehabilitation decision matrix was developed, as presented in Table 16.

**Table 16:** PCI based maintenance and rehabilitation decision matrix.

PCI band	Condition	Typical strategy	Representative treatments
> 85	Excellent	Preventive	Routine crack sealing, monitoring
70–85	Good	Preventive	Crack sealing, fog/rejuvenating seal, surface treatment
55–70	Fair	Light corrective	Thin overlay, slurry/micro-surfacing, localized patching
40–55	Poor	Major corrective	Mill-and-overlay, structural overlay, edge repair
25–40	Very Poor	Rehabilitation	Deep patching + thick overlay or partial reconstruction
< 25	Failed	Reconstruction	Full-depth reconstruction of base and surface

### Section-by-section management strategy

The recommended maintenance strategy was developed by integrating the current PCI condition, observed distress progression, and predicted future deterioration of each roadway section. The proposed interventions were prioritized according to the severity of pavement deterioration and the dominant distress mechanisms identified from the TLS observations.

The highest priority should be assigned to Sections “Study area, instruments and data acquisition” and “Predictive modeling framework”, where the pavement condition has already reached critical levels. Both sections exhibited the lowest PCI values and the highest deterioration rates during the monitoring period. Section “Study area, instruments and data acquisition” showed a combination of severe rutting, increasing pothole development, slippage cracking, and lane–shoulder drop-off, indicating advanced structural deterioration rather than isolated surface defects. Therefore, preventive or surface-based treatments are unlikely to provide a sustainable solution, and full-depth reconstruction is recommended. Similarly, Section “Predictive modeling framework” exhibited fatigue cracking, pothole formation, and extensive patching, suggesting significant pavement weakening. A rehabilitation strategy involving deep patching followed by a structural overlay is recommended, with reconstruction considered if further deterioration occurs.

An intermediate management priority is represented by Sections “Results and discussion” and “Decision-support framework for pavement maintenance management”. Despite not yet falling into the failed condition category, these parts' declining trends show that prompt remedial action is required. Large scale block cracking and raveling, along with yearly PCI losses of roughly 2.3–2.5 points per year, indicate that postponing repair could hasten pavement deterioration. In order to improve pavement performance, restrict moisture penetration, and stop the emergence of more serious distresses like potholes, mill-and-overlay or structural overlay treatments are advised.

Sections “Introduction”, “Research methodology and plan”, “Integrated data processing and analysis”, and “Forecasting and risk classification of pavement condition”, on the other hand, are still in comparatively good shape and fall within the Good to Fair PCI classifications. These areas offer a chance for preventive maintenance, where comparatively inexpensive repairs can maintain the current state of the pavement and increase its lifespan. To stop early-stage deterioration and postpone the switch to more costly rehabilitation therapies, crack sealing, fog or renewing sealants, and localized patching are advised.

### Prioritization logic

The maintenance prioritization framework was created by combining distress increase rates, statistical correlations to PCI, future condition projections, and the PCI-based treatment matrix. Two core distress groups were identified as useful indicators to help guide intervention decisions. Rapid pothole development is an early indicator of localized pavement deterioration; thus, sections with accelerated pothole growth, particularly Sections “Study area, instruments and data acquisition”, “Introduction”, “Predictive modeling framework”, and “Research methodology and plan”, should get increased inspection and timely correction activity.

In addition, rutting and lane–shoulder drop-off showed the strongest negative relationships with PCI, indicating their importance as indicators of structural pavement deterioration. These distresses require structural treatments rather than surface-level repairs to prevent further condition decline.

By combining the 2030 PCI forecasts (Table 15) with the maintenance decision matrix (Table 16), a prioritized multi-year maintenance plan can be established. Sections ‘Study area, instruments and data acquisition” and “Predictive modeling framework” should receive immediate rehabilitation, Sections “Results and discussion” and “Decision-support framework for pavement maintenance management” should be considered for corrective overlay treatments, and the remaining sections should be maintained through preventive treatments.

The proposed TLS-based framework supports a transition from reactive repair toward condition-based maintenance by identifying deterioration trends before critical pavement failure occurs, thereby improving maintenance planning and resource allocation.

## Conclusion

This study investigated the application of multi-temporal terrestrial laser scanning (TLS) for pavement distress assessment, deterioration analysis and condition forecasting of a flexible pavement roadway. The study was conducted on a 20.1 km roadway in Nile Delta, Egypt, using four TLS observations epochs collected between November 2022 and March 2025. The results confirmed that TLS can provide accurate and repeatable measurements of pavement surface distresses, including distress types that are difficult to quantify using conventional visual inspection methods. The monitored roadway sections showed continuous deterioration during the study period, with PCI reductions ranging from 5.4 to 45%. Potholes exhibited the highest deterioration rate, reaching approximately 45% per year, indicating their importance as an early indicator of localized pavement degradation. The regression analysis showed a significant relationship between pavement distress characteristics and PCI, with the OLS model achieving R^2^ = 0.833 and adjusted R^2^ = 0.785. Rutting, lane–shoulder drop-off, raveling, and linear cracking were identified as the most influential distress variables affecting pavement condition. The comparison of prediction models showed that tree-based machine-learning models achieved high accuracy within the investigated roadway sections, with R^2^ values ranging from 0.972 to 0.984 and RMSE values below 4 PCI points under leave-one-out validation. However, their performance decreased under leave-one-section-out validation due to the limited number of independent pavement sections. The temporal PCI deterioration models provided a more reliable approach for forecasting future pavement conditions, achieving a mean R^2^ of 0.91. The forecasts indicated that Sections “Study area, instruments and data acquisition” and “Predictive modeling framework” are expected to reach critical condition levels before 2030 if current deterioration trends continue. The findings of this study show that combining TLS-based distress measurements, PCI evaluation, statistical analysis, and deterioration forecasting can improve pavement condition assessment and support maintenance prioritization. The proposed approach provides transportation agencies with quantitative information for identifying critical sections and selecting appropriate maintenance actions.

## Recommendations and future studies

The results of this study highlight the value of integrating multi-temporal terrestrial laser scanning (TLS) observations with pavement condition evaluation for improving pavement monitoring and maintenance planning. TLS-based surveys are recommended as a complementary approach to conventional pavement inspection, particularly for road sections experiencing heavy traffic loading or progressive deterioration, as they provide quantitative information on distress development over time. Maintenance decisions should consider both the current PCI level and the deterioration rate of individual distress types. Distresses such as potholes, rutting, and lane–shoulder drop-off should receive priority because of their strong association with pavement deterioration. Therefore, incorporating distress progression analysis and condition forecasting into pavement management programs can support timely interventions and improve resource allocation. Future studies should expand TLS-based monitoring to larger and more diverse roadway networks covering different pavement structures, traffic conditions, and climatic environments. Increasing the number of monitored sections and observation periods will improve the reliability of deterioration models. Future research should also integrate TLS-derived distress measurements with additional factors, including traffic loading, structural properties, drainage conditions, and environmental variables, to enhance pavement performance prediction and maintenance planning. The relatively small dataset of 32 section–epoch observations represents a limitation that may affect model stability and increase the risk of overfitting. To mitigate this issue, leave-one-out cross-validation and leave-one-section-out cross-validation were used to assess model performance. Nevertheless, the predictive results should be interpreted primarily within the context of the investigated roadway and observation period rather than as universally generalizable models. Future studies should incorporate larger datasets covering additional roadway sections, longer monitoring periods, and diverse pavement conditions to further validate the generalization capability of the proposed models. Future studies should provide detailed machine-learning implementation settings, including model parameters, training procedures, hyperparameter tuning, and optimization strategies, to further enhance the reproducibility and generalization of the proposed approach. Future research should combine TLS-based surface distress assessment with direct structural evaluation methods, such as pavement deflection testing, to better identify surface deterioration from underlying structural deterioration and make more reliable maintenance recommendations. Although ASTM D6433-24 was released after the data collection period, verification of the study results using the updated standard showed no changes affecting the reported findings. Future studies will adopt ASTM D6433-24 for subsequent pavement-condition assessments.

## Data Availability

The data that support the findings of this study are available from the corresponding author upon reasonable request.
